# Site-Specific Structural Changes in Long-Term-Stressed Monoclonal Antibody Revealed with DEPC Covalent-Labeling and Quantitative Mass Spectrometry

**DOI:** 10.3390/ph16101418

**Published:** 2023-10-05

**Authors:** Manasi Gaikwad, Florian Richter, Rabea Götz, Aline Dörrbaum, Lena Schumacher, Jason Tonillo, Christian Frech, Roland Kellner, Carsten Hopf

**Affiliations:** 1 Center for Mass Spectrometry and Optical Spectroscopy (CeMOS), Mannheim University of Applied Sciences, Paul-Wittsack-Str. 10, 68163 Mannheim, Germany; m.gaikwad@hs-mannheim.de (M.G.); f.richter@hs-mannheim.de (F.R.);; 2 Merck Healthcare KGaA, ADCs & Targeted NBE Therapeutics, Frankfurter Str. 250, 64293 Darmstadt, Germany; 3 Faculty of Biotechnology, Mannheim University of Applied Sciences, Paul-Wittsack-Str. 10, 68163 Mannheim, Germany; 4 Medical Faculty, Heidelberg University, 69117 Heidelberg, Germany

**Keywords:** diethyl pyrocarbonate labeling, monoclonal antibody, heat stress, long-term storage, site-specific labeling

## Abstract

Studies of structural changes in mAbs under forced stress and storage conditions are essential for the recognition of degradation hotspots, which can be further remodeled to improve the stability of the respective protein. Herein, we used diethyl pyrocarbonate (DEPC)-based covalent labeling mass spectrometry (CL-MS) to assess structural changes in a model mAb (SILuMAb). Structural changes in the heat-stressed mAb samples were confirmed at specific amino acid positions from the DEPC label mass seen in the fragment ion mass spectrum. The degree of structural change was also quantified by increased or decreased DEPC labeling at specific sites; an increase or decrease indicated an unfolded or aggregated state of the mAb, respectively. Strikingly, for heat-stressed SILuMAb samples, an aggregation-prone area was identified in the CDR region. In the case of longterm stress, the structural consequences for SILuMAb samples stored for up to two years at 2–8 °C were studied with SEC-UV and DEPC-based CL-MS. While SEC-UV analysis only indicated fragmentation of SILuMAb, DEPC-based CL-MS analysis further pinpointed the finding to structural disturbances of disulfide bonds at specific cysteines. This emphasized the utility of DEPC CL-MS for studying disulfide rearrangement. Taken together, our data suggests that DEPC CL-MS can complement more technically challenging methods in the evaluation of the structural stability of mAbs.

## 1. Introduction

Monoclonal antibodies (mAbs) are susceptible to structural changes, such as aggregation during storage or physiochemical stress conditions, which are ultimately a challenge to the immunogenic safety of the drug [1]. Before the deployment of mAbs in the market, European and US regulatory guidelines demand stress testing of mAbs to study associated mAb degradation pathways and ultimately to derive information on the structure–function relationship of mAbs [2]. The results of stability tests of mAbs or those of other proteins are, however, dictated by the sensitivity and accuracy of the analytical techniques used for the assessment [3]. Thus, the choice of analytical method used for the structural characterization of mAbs is of immense significance. Especially with the growing speed of entry of mAbs into the market, the structural characterization methods of mAbs need to be not only sensitive and accurate but also fast and efficient.

Several techniques are used in pharmaceutical research and development for testing a protein’s structural stability [4,5]. As compared to more sophisticated biophysical methods, such as NMR or X-ray crystallography, use of liquid chromatography–mass spectrometry (LC-MS) for structural characterization of mAbs has increased. For instance, intact-protein analysis by native mass spectrometry is now being used to report the structural homogeneity of a mAb and its interaction with other biomolecules [6,7]. Hydrogen-deuterium exchange MS (HDX-MS) is arguably the most widely used technique to probe structural changes in a mAb, studying unfolding events and epitope mapping [8]. One key drawback limiting the use of HDX-MS is the rapid back-exchange of deuterium, which demands specialized equipment for fast experimentation at low temperatures [9,10]. An alternative technique of potential use is covalent labeling MS (CL-MS), wherein the structural changes in proteins can be interpreted from modifications of aa side chains by small- or medium-mass chemical reagents [11]. In CL-MS-based structural protein analysis, on one end, the state of the protein itself, its primary sequence, tertiary structure, and the chemical microenvironment are important factors in labeling [12,13,14]. On the other end, the accessibility of potential sites for the labeling reagent, and thereby its detection capability, is equally significant. Fast photochemical oxidation of proteins (FPOP) is one example of the CL-MS technique, which uses laser-induced hydroxyl radicals as a labeling reagent. These hydroxyl radical labels can be detected by a specific mass shift via LC-MS, ultimately capturing snapshots of a protein’s conformational state [15,16]. However, the application of FPOP is limited due to specific buffer needs, costs, high safety requirements of laser equipment, and complex datasets requiring specialized data analysis [16].

DEPC-based CL-MS, in contrast, offers straightforward labeling protocols, utilization of common LC-MS setup, reactivity towards multiple aa side chains, and comparatively simple data interpretation [17]. Unlike HDX-MS, which captures changes in protein dynamics as well as solvent accessibility, changes in DEPC labeling across different conditions are expected to primarily indicate changes in solvent-accessible surface areas (SASA) of the protein [18]. An average of 30% of the protein structure can be probed by a DEPC labeling strategy in CL-MS [19]. Side chains of Lys and His are preferentially labeled by DEPC. Besides these, Ser, Thr, and Tyr can also be DEPC-labeled, but show a lower correlation to changes in protein SASA [14,19]. DEPC reacting with free Cys has also been reported [19,20]. DEPC reaction with these aa side chains results in the MS-detectable addition of carboethoxy groups. In the case of excess DEPC reacting with His, three other reaction products have been described, namely formyl-carboethoxyhistidine (formyl-CEt-His), 1,3-dicarboethoxyhistidine (di-CEt-His), and urethane-carboethoxyhistidine [21]. 

The application of DEPC CL-MS has proven useful for the study of SASA in proteins or protein complexes, as well as for epitope mapping. DEPC CL-MS is particularly beneficial for studying protein topology, as DEPC labeling preferentially occurs in the solvent-exposed regions of proteins [19,22,23,24,25,26].

In this study, the application of DEPC CL-MS was explored to study structural changes in mAb subjected to stressed conditions. Initially, we focused on the structural stability of the mAb samples subjected to heat stress. Most published research suggests that heat-stressing mAbs at 90 °C causes irreversible structural changes, disturbing its antigen-binding capacity [27,28,29,30]. However, the initiation of site-specific structural changes in an intact IgG stressed below its melting point is also a relevant part of mAb stress testing. This was explored in our study with the application of limited heat stress (65 °C) for 1 h. Another important aspect of analytical testing in mAb development that was focused on in our study was the shelf-life of non-formulated drug substance. The long-term storage testing guidelines in ICH Topic Q1 A (R2) demand a testing duration of >1 year [2], and therefore, we assessed mAb samples that had been stored for up to 2 years. Moreover, there are indications that the thermal stability of long-term-stored mAbs can be compromised [31]. Therefore, the structural integrity of long-term-refrigerated (“non-formulated, aged”) SILuMAb, as well as its thermal stability, was assessed separately using DEPC-based CL-MS.

## 2. Results

**Figure 1:**
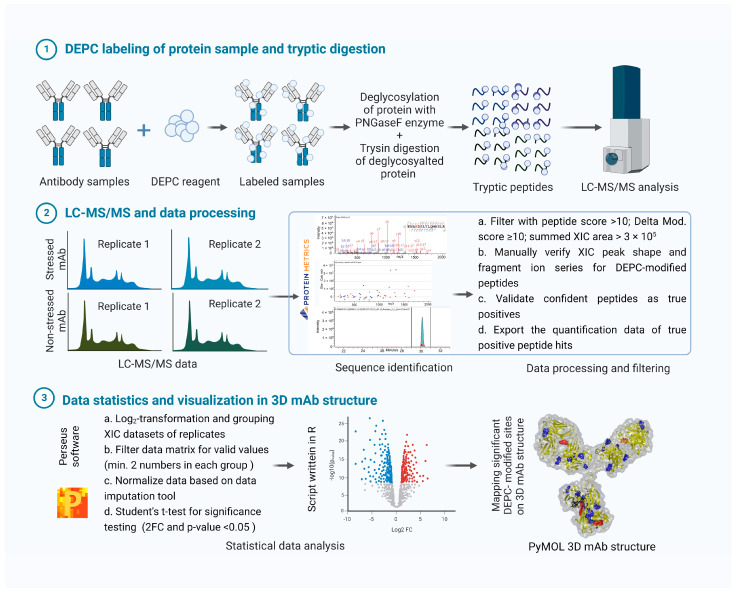
Workflow for DEPC labeling of protein and detailed bottom-up MS analysis: DEPC labeling of intact mAbs is followed by deglycosylation and tryptic digestion to generate peptides for bottom-up analysis. LC-MS/MS data are processed in Byos software version: v4.5 (peptide identification and quantification). Peptide hits are visually inspected to confirm correct identification. Downstream statistical data analysis is performed in Perseus (Student’s *t*-test), and the results are visualized in a scattered volcano plot, showing significant changes in the intensity of certain DEPC-modified peptides. Significant DEPC-modified sites are further represented on a PyMOL-generated 3D mAb model. (Figure generated with licensed version of Biorender).

Structural changes in DEPC-labeled mAb samples can be studied at the intact protein level as well as the peptide level. As the sensitivity and resolution of intact-protein MS is low for large molecules such as mAbs, bottom-up LC-MS/MS analysis is used for detailed structural characterization in our study (Figure 1).

### 2.1. Reproducibility of DEPC Labeling

Initially, we evaluated the inter-day reproducibility of the experimental setup using freshly thawed SILuMAb samples. Three independent labeling experiments were performed on three different days. An unlabeled SILuMAb sample was used as a negative control for each of the three experimentation days.

**Figure 2:**
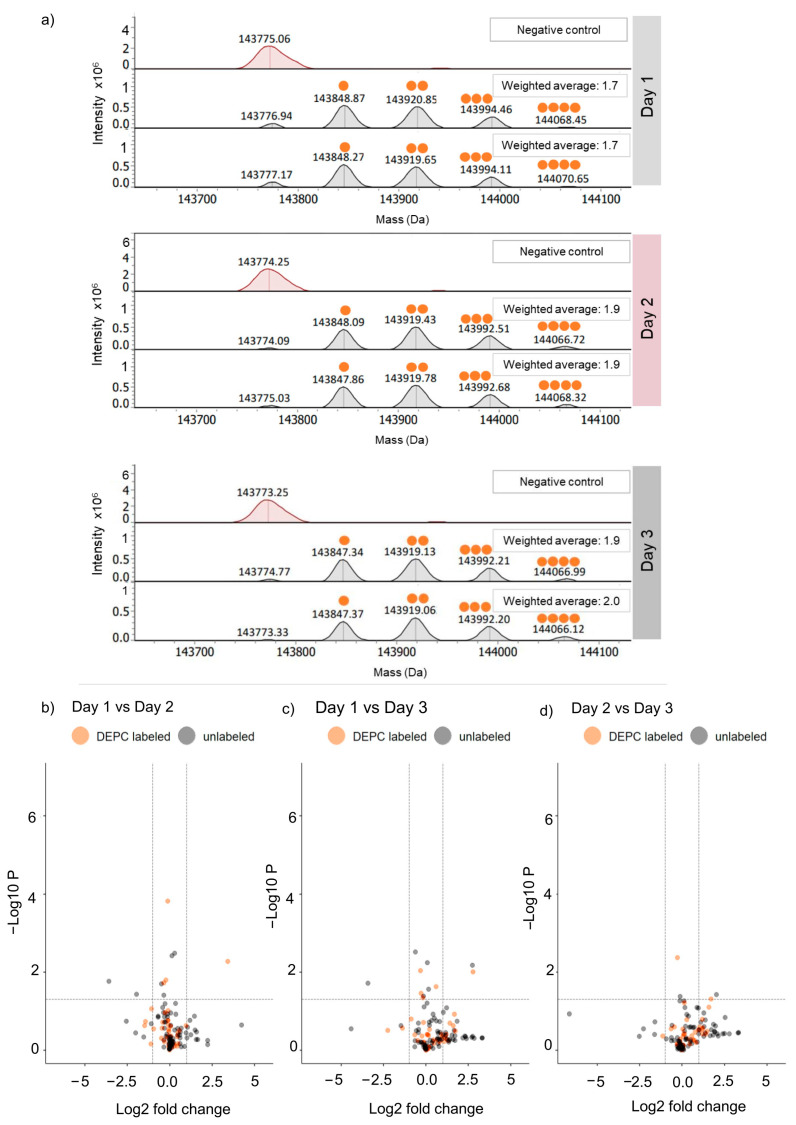
Inter-day reproducibility experiment performed on three different days: (**a**) Deconvoluted spectrum of each sample along with mass annotations (in Da) are displayed. The unlabeled mAb as a control for each respective day is highlighted in maroon. DEPC-labeled mAb replicates (n = 2 per day) are highlighted in grey, and the orange dots represent the number of DEPC labels. The reproducibility of the labeling protocol is reflected in similar weighted average labels obtained across days 1 to 3. Inter-day reproducibility of DEPC labeling assessed with bottom-up MS across days 1 and 2 (**b**), days 1 and 3 (**c**), and days 2 and 3 (**d**), wherein each dot in the scatter plot depicts a tryptic peptide with a specific − Log10 *p*-value (with *p* < 0.05 defined as significance level indicated by a dotted line) for differences (expressed as log2 fold-change with a 2-fold change defined as significance level) in intensity. Orange dots represent the DEPC-labeled peptides, while grey dots are unlabeled peptides.

On the intact-protein level, analysis of the unlabeled SILuMAb as control (maroon-colored deconvoluted spectrum in Figure 2) revealed an average mass of 143,774.25 Da (±6 ppm) for the deglycosylated SILuMAb. DEPC labeling led to a characteristic mass shift of 72.02 Da per label. The deconvoluted mass spectra of labeled samples showed five peaks corresponding to 0 to 4 DEPC-label incorporating SILuMAb masses indicated with orange overhead dots (Figure 2). The labeling pattern was similar for all independent replicates. The average number of labels per protein was calculated as the average label incorporation weighted by the signal intensities of the corresponding MS peaks, as described in the formula in Section 4.4.3. Similar average labels (ranging from 1.7 to 2.0) were observed for all experimental replicates, demonstrating the reproducibility of the labeling procedure itself. It should be acknowledged that, in intact-protein MS analysis, lower detection sensitivity for higher DEPC-incorporated species maybe due to the lower dynamic range of the instrument.

The independent labeling replicates were also subjected to the bottom-up MS analysis. Tryptic peptides were identified with between 0 and 3 missed cleavages in our CL-MS study. High sequence coverage levels of 97.56% and 96.33% were achieved for the heavy chain (HC) and light chain (LC), respectively. Volcano scatter plots were obtained from the student’s *t*-test on the total quantitative dataset (Figure 2b–d) of all labeled and non-labeled peptides. In the volcano plot, grey-colored dots represent peptides with no DEPC label, while orange-colored dots are peptides with DEPC modification at specific amino acidss. The criterion for reproducibility was peptides showing no significant change; i.e., no peptides with *p* < 0.05 and less than a two-fold change in intensity were included. Except for one DEPC-labeled peptide detected in the day 1 and day 2 experiments (Figure 2b,c—orange dots), all DEPC-labeled peptides were inside the dotted lines representing boundaries for significant changes. In other words, DEPC labeling and its detection with the current protocol was reproducible and did not vary for mAb samples assessed in a similar structural state.

### 2.2. Heat-Stress-Induced Structural Changes in SILuMAb

**Figure 3:**
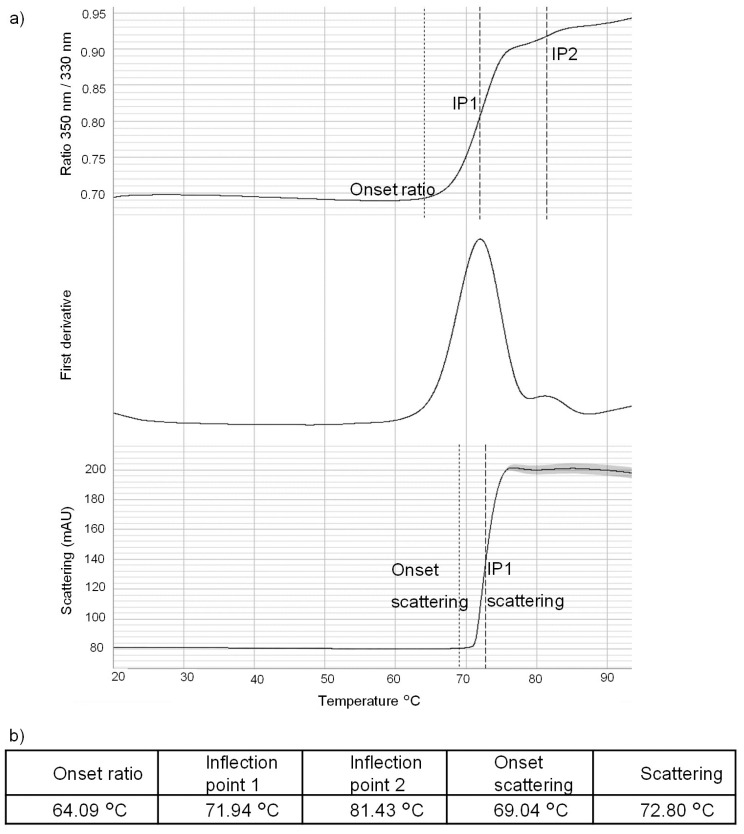
NanoDSF analysis of SILuMAb. (**a**) Thermogram of SILuMAb showing temperature for onset ratio, inflection point 1 and inflection point 2 respectively (top), first derivative of the thermogram (center), and light scattering thermogram showing temperature for onset scattering and scattering respectively (bottom). (**b**) Temperature values of the onsets and inflection points are noted in the table.

One of the major interests of this study was assessing structural changes induced by heat stress. First, the SILuMAb was studied by nano differential scanning fluorimetry (nanoDSF) to find its melting temperature, wherein temperature was increased from 20 °C to 95 °C within 75 min, and the resulting structural change in the SILuMAb was evaluated with nanoDSF (Figure 3). The onset of unfolding was noted at 64.09 °C. This marked the lowest temperature at which the SILuMAb underwent conformational changes. At 71.94 °C, 50% of the least thermally stable domain was unfolded (inflection point 1), and at 81.43 °C, 50% of the second-least thermally stable domain was unfolded (inflection point 2). The onset of aggregation was determined at 69.04 °C (onset scattering), while 72.80 °C was the temperature at which 50% of the mAb was aggregated (scattering). As the initial structural changes were noted at around 64.09 °C, 65 °C was chosen as a heat-stress condition to induce stress-related structural changes.

**Figure 4:**
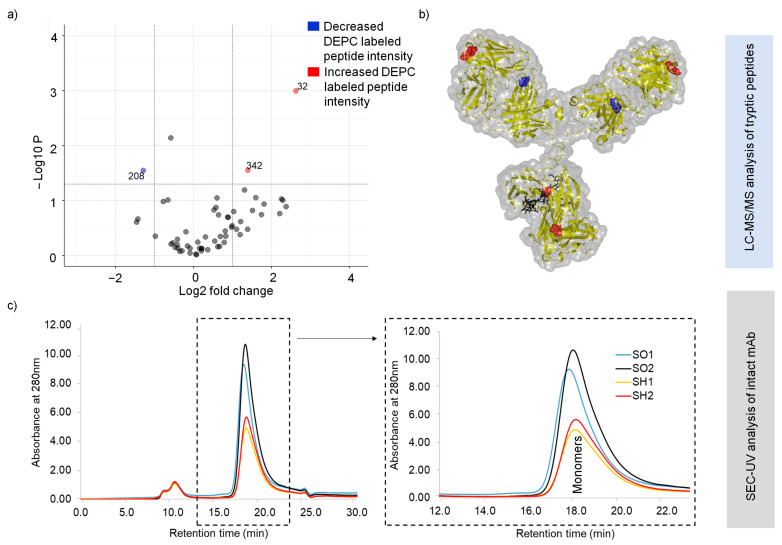
Sites of structural changes in a heat-stressed versus a non-heat-stressed SILuMAb. (**a**) Scatter plot; (**b**) 3D SILuMAb structure. The sites of significantly increased DEPC label incorporation in the heat-stressed mAb are presented in red (Tyr32 and Lys342). Blue annotation represents the aa positions with decreased DEPC labeling across the replicates of the heat-stressed mAb (His208). (**c**) SEC–UV analysis of heat-stressed (yellow and red) and non-heat-stressed (blue and black) SILuMAb samples. Replicates of the latter showed a prominent monomeric peak at 16–18 min. Replicates of the heat-stressed mAb displayed decreased intensity than that of the monomeric peak.

To identify the initiation of heat-stress-related structural changes with DEPC CL-MS, the non-stressed SILuMAb (SO) sample set was compared against the heat-stressed (65 °C for 1 h) sample set (SH). The experimental setup for DEPC labeling was as specified in Section 4.1 (i). Sequence coverage of 100% was obtained for both HC and LC in bottom-up MS analysis. Forty-seven DEPC sites (Supplementary Table S1) could be quantified for the SO and SH sample sets, respectively. Lys (28 sites) was the preferentially modified aa among the 47 labeled sites detected. Amongst all labeled sites, only three sites on the HC, namely Tyr32, His208, and Lys342, showed significant changes (*p* < 0.05 and two-fold difference in labeled peptide intensity) in heat-stressed mAb ssamples (Figure 4a). These positions are represented in the 3D SILuMAb structure (Figure 4b).

The decreased DEPC labeling for heat-stressed SILuMAb samples at His208 might be due to the local compaction of the mAb molecule induced by the heat stress. In the case of Tyr32 and Lys342, an increase in DEPC labeling was observed in comparison to the non-heat-stressed sample. Notably, Tyr32 was located within the CDR-1 region (complementary determining region lying within the variable region of the heavy chain).

Supporting intact-protein analysis by SEC-UV of both heat-stressed and non-heat-stressed SILuMAb samples (Figure 4c) indicated no aggregates (which would elute earlier than the monomer), or fragments (which would elute later than the monomer). However, it should be noted that partially unfolded mAb molecules in the heat-stressed samples would not be captured as separate peaks in SEC-UV analysis, due to its low-resolution capacity. The peak at 10 min was seen to be present in blank injection as well as standard mAb injection (Supplementary Figure S2), and was thus inferred to be a column-specific contaminant unrelated to the mAb samples injected. In both cases, only one protein species eluted at a retention time of 18 min (Figure 4c). This peak can be identified as a SILuMAb monomer related to the peaks seen in standard mAb SEC-UV analysis with known retention times of monomers (Supplementary Figure S2). The monomeric peak from the heat-stressed SILuMAb (SH), however, displayed lower intensity, hinting that protein loss might be caused by the precipitation of partially unfolded/semi-aggregated heat-stressed mAbs on the walls of sample vial, or by on-column precipitation, which was invisible in our SEC-UV analysis.

### 2.3. Structural Changes in Heat-Stressed, One-Year-Stored SILuMAb

**Figure 5:**
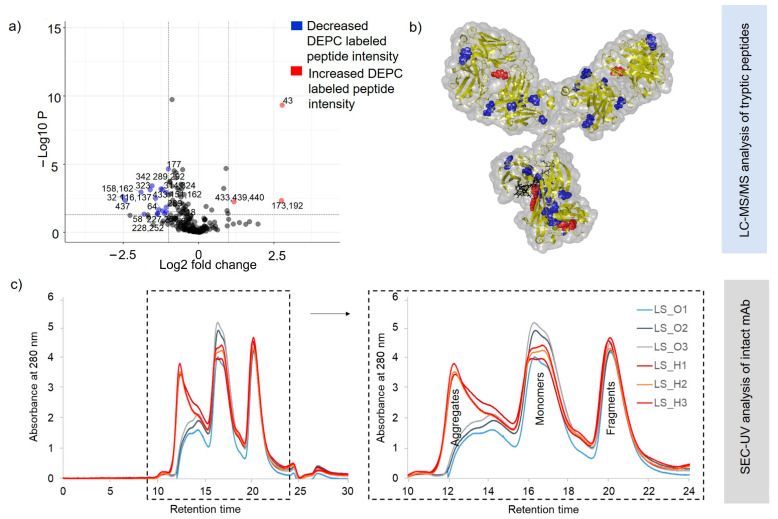
Sites of structural changes in a non-stressed, one-year-stored SILuMAb and a heat-stressed one-year-stored SILuMAb. (**a**) Scatter plot; (**b**) 3D SILuMAb structure. The sites of significantly increased DEPC labeling in heat-stressed one-year stored mAb samples (LS_H) are presented in red. The blue annotations represent the aa positions with decreased DEPC labels across replicates of LS_H mAb; (**c**) SEC–UV analysis of (3×) heat-stressed one-year-stored mAbs (LS_H1, LS_H2, LS_H3) and (3×) one-year-stored non-heat-stressed mAb samples (LS_O1, LS_O2, LS_O3). The latter replicates show a prominent peak at 16–18 min, indicative of monomer, a peak at 12–14 min, representing aggregated mAbs, and a peak at 20–22 min, indicating fragmented mAbs.

Differential scanning calorimetry (DSC) studies have previously reported that the thermal stability of long-term-stored mAbs is compromised [32]. We examined the effects of heat stressing a SILuMAb that had been stored for one year (at 2–8 °C). The intent was to capture the worst cases of structural changes in the mAb sample with DEPC CL-MS. The experimental setup for DEPC labeling was as specified in Section 4.1 (ii). Two sets of mAb samples were considered herein: non-heat-stressed, one-year-stored mAb (LS_O) and heat-stressed (65 °C for 1 h), one-year-stored mAb (LS_H). Analysis of the LC-MS/MS datasets resulted in the detection of 119 aa positions with DEPC modifications (Supplementary Table S2), wherein the DEPC label was confirmed with MS/MS fragment ion series. Among the 119 sites, 21 aa positions showed significant quantitative differences in DEPC labeling (with *p* < 0.05 and two-fold intensity difference) across the LS_O and LS_H sample sets (Figure 5a). These differentially labeled positions are visualized in a model 3D SILuMAb structure (Figure 5b). Reduced DEPC labeling suggested that the respective regions most likely were not accessible for DEPC incorporation after heat stress. Thus, areas marked with blue dots might be aggregation-prone in heat-stressed, one-year-stored SILuMAb samples. Multiple aggregation-prone spots were identified in the LS_H mAb (Figure 5b) in contrast to the SH mAb sample, which was not refrigerated for one year (Figure 4b), and where only one site showed a reduced DEPC label. Most regions showing changes in DEPC-labeled peptide intensity (blue dots) for LS_H samples were distributed in the Fab region. On the other hand, the red dots indicate the aa positions with increased DEPC intensity for the LS_H sample set, perhaps indicating the intermediate unfolded state of the SILuMAb before aggregation.

Supplementary SEC-UV analysis of the LS_O and LS_H samples after DEPC labeling (but before tryptic digestion) led to the following observations (Figure 5c): (1) The LS_O (blue, black, grey color) chromatograms clearly showed three peaks—an aggregate peak indicating two overlapping species at 12–14 min, a putative monomer peak at 16-18 min, and a peak presumably representing fragmented mAbs at 20–22 min. (2) By comparison, the heat-stressed, one-year-stored mAb samples (LS_H) displayed increased aggregation, reflected in the increased height of the aggregate peak and slight time shift to 12–14 min. A possible explanation of this peak could be the multimerization of preexisting unfolded or fragmented mAb species, upon the application of thermal stress. These results agree with the results of bottom-up MS analysis, wherein almost 18 aa positions (blue dots) hinted at possible aggregation-prone areas. (3) In the LS_H sample set, the shape and height of the monomer peak were altered, indicating compromised monomer in comparison to the LS_O sample set. (4) Peak height and area of the “fragment” peak 3 were unchanged by heat stress. This indicates that the non-stressed one-year-stored mAb sample had pre-existing mAb degradation fragments and a level of aggregation.

### 2.4. Intactness of Disulfide Bonds in Long-Term-Stored SILuMAb

**Figure 6:**
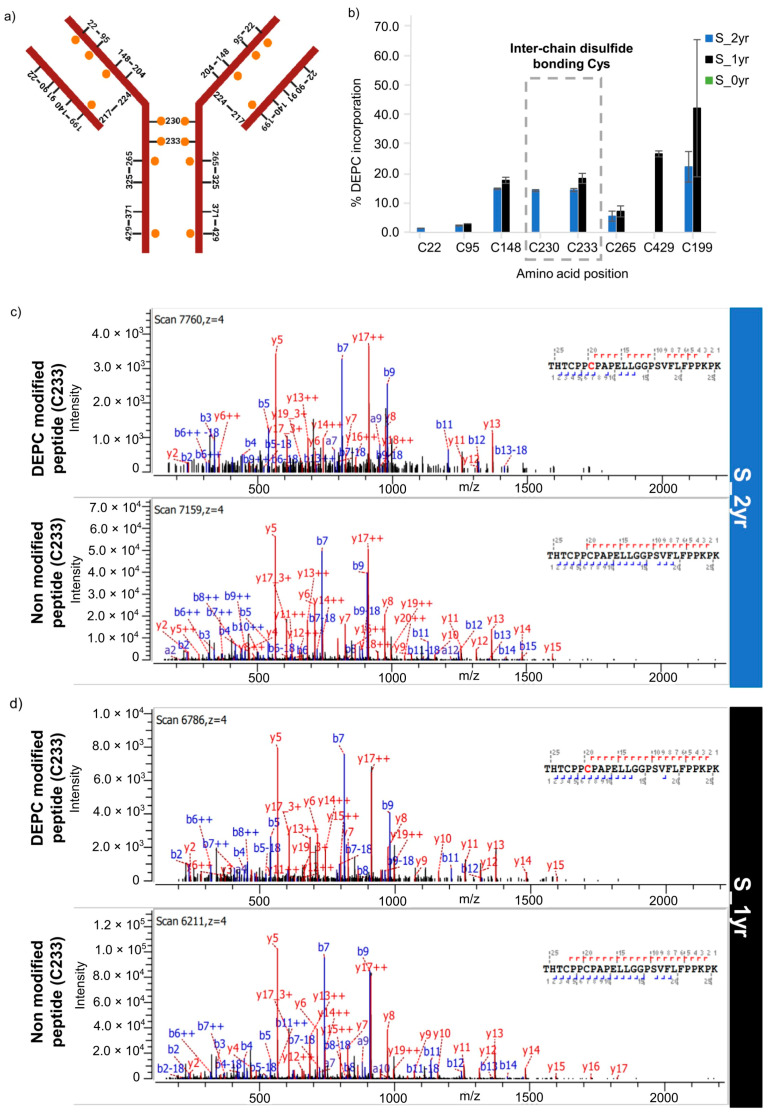
Sites with disrupted disulfide bonding in a long-term-stored (S_2yr and S_1yr) SILuMAb in comparison to a fresh, non-stored SILuMAb. (**a**) Schematic representation of disulfide bonding amongst the cysteines of SILuMAb. Orange dots highlight DEPC-modified Cys detected in long-term-stored (S_1yr and S_2yr) mAb samples. (**b**) DEPC label average % (from 4 replicates) at specific Cys positions is represented in a bar chart along with standard deviation. The blue bars represent S_2yr samples and black bars represent S_1yr samples. No DEPC incorporations were visible at the cysteines of the fresh mAb sample set; thus, no green bars are seen in the bar chart. (**c**) From top to bottom panels for sample S_2yr: fragment ion spectra of peptide containing Cys233 and the corresponding unmodified peptide with no DEPC incorporation at Cys233. (**d**) As in (**c**), but for sample S_1yr.

We aimed to understand the preformed aggregate peak and the fragment peak in the long-term-stored SILuMAb sample, and assumed that a certain instability of the mAb structure might be related to the disulfide bond patterns within the respective sample sets. To investigate the stability of the once-thawed and long-term-refrigerated antibody sample in more detail, we assessed the intactness of known disulfide bonds within the mAb by studying the DEPC-labeled Cys. The background was that DEPC would only be reactive towards free Cys in the mAb sample. Three different sets of SILuMAb samples were used for this experiment: mAb refrigerated for 2 yrs (S_2yr), mAb refrigerated for 1 yr (S_1yr), and fresh mAb (S_0yr). The experimental setup for DEPC labeling was as specified in Section 4.1 (iii). Among 15 Cys sites on SILuMAb, 8 sites (seven on HC and one on LC) were confirmed to have DEPC modification in mAb samples refrigerated for 1 yr and 2 yrs (Figure 6a,b), but not in the freshly thawed, non-stored mAb sample (S_0yr). Except for Cys199, the other seven Cys sites in Figure 6b refer to the position on HC (details in Supplementary Table S3). For the 2-year-old sample, DEPC was detected at all positions except Cys429. For most DEPC-labeled Cys sites detected herein, DEPC % in the 2-year-old and 1-year-old mAb samples was comparable. We did not detect DEPC labels on LC Cys217 and HC Cys224, which linked the two chains together (Figure 6a). This result implies that HC and LC probably stayed together during the storage time frame. Furthermore, we detected Cys148 on HC but missed its counterpart intrachain bonding Cys204 on HC, which suggested disulfide scrambling. Similarly, on the LC, we detected Cys199 but missed Cys140, which should ideally be involved in a disulfide bond. Cys199 on LC was detected with quite high error bars within the 2 yr sample, but not in the 1 yr sample, which showed that disulfide involving Cys199 might have been compromised by different processes, or may have been prone to oxidation after being released from its natural disulfide state in the S_2yr sample. The point of special interest is that DEPC was also detected at Cys233 on HC, a cysteine involved in interchain disulfide bonding. DEPC incorporation at Cys233 for S_2yr and S_1yr can be seen from the fragment ion spectra (Figure 6c,d). This finding pointed to the potential disassembly of the heavy chains that are supposed to be held together in the antibody. Besides weakening interchain linkage, this could potentially open hydrophobic surfaces for aggregation. While the other Cys involved in disulfide bonding (Cys230) showed almost 15% DEPC for the 2-year-old mAb sample, a label at the same position was not detected at substantial levels in the 1-year-old sample (Figure 6b). DEPC incorporation at these Cys sites indicates that disulfide bonds, and thereby the overall mAb structure, were compromised due to their storage at 2–8 °C before the labeling experiment.

**Figure 7:**
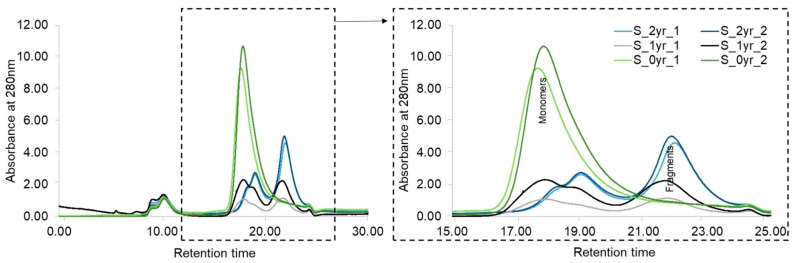
SEC–UV analysis of (2× S_2yr, (2×) S_1yr, and (2×) S_0yr mAb samples. Chromatograms (duplicates) of the freshly thawed S_0yr mAbs (light and dark green) showed a prominent “monomeric” peak at 17–19 min. Those of the S_1yr mAb samples (black and grey) showed two shoulder peaks at 17–19 min and a putative fragment peak of the same height at 21–23 min. Chromatograms of the S_2yr mAb samples (light and dark blue) showed two small shoulder peaks at 17–19 min and a prominent fragment peak of double the height at 21–23 min.

To confirm this finding from bottom-up MS analysis, the same long-term-stored and DEPC-labeled mAb samples were analyzed at the intact-protein level with SEC-UV (Figure 7). In comparison to standard GammaNorm mAb (Supplementary Figure S2), the RT of the monomer was confirmed at 17–20 min. The monomer was abundant in the S_0yr sample, while S_2yr and S_1yr samples showed 5 times less intensity for the putative monomer peak (Figure 7). Moreover, the “monomer” peak of S_2yr and S_1yr was divided into two shoulder peaks, indicating two or more co-eluting mAb fragments that were non-covalently bound together, or fragments bound with disulfide rearrangements.

On the other hand, while there was no “fragment” peak in the S_0yr sample, an evident peak at 21–23 min for the S_2yr and S_1yr samples indicated that the long-term-stored mAb samples had high amounts of pre-existing fragments. Moreover, the intensity of the “fragment” peak for the older S_2yr (blue chromatogram) sample was higher than for S_1yr (black chromatogram). These SEC-UV results were clear evidence that except for the fresh sample S_0yr, both the 1-year- and 2-year-refrigerated mAb samples were highly degraded, with minimal monomer amounts remaining in the sample. These SEC-UV results showing no presence of monomer in the S_2yr and S_1yr samples were further confirmed when the same samples were analyzed using RPLC-MS (Supplementary Figure S3), yielding no monomer mass detection.

## 3. Discussion

Forced degradation studies and the associated structural protein characterization constitute an inevitable part of biopharmaceutical development. Industry experts have pointed out the common analytical techniques used for product characterization [33], but CL-MS is not listed among the most common analytical characterization techniques yet. However, it is emerging as a complementary technique to HDX-MS for structural mAb analysis, as the latter requires more sophisticated technical equipment in the laboratory. It must be noted, however, that unlike HDX-MS, which uses small-sized deuterium labels, the labeling of proteins with medium -sized reagents, such as DEPC in CL-MS, can potentially induce artificial structural changes in proteins. Therefore, DEPC-based CL-MS requires definite experimental design and the study of its reaction kinetics [13,34]. Reaction kinetics testing of DEPC labeling was performed as a part of this study, and the relevant summary is mentioned as part of the Supplementary information. Briefly, the concern was that above a threshold DEPC concentration X, the reagent itself may cause the unfolding of the mAb. This could be observed as a kink at concentration X in an otherwise linear plot of DEPC concentration versus the ratio of summed peak areas of DEPC-modified peptides and total peptides (Supplementary Figure S1). Such a break in linearity was reported at a six-fold excess of DEPC, meaning that above this concentration, the reagent itself may affect the protein structure [34]. However, in our study (Supplementary Figure S1), there was no such break in linearity at 6× molar DEPC in a plot analyzing multiple SILuMAb peptides. Instead, linearity extended even beyond a 10× molar excess of DEPC. Based on the observation of DEPC reaction kinetics for multiple proteins, a DEPC concentration of 10× molar excess or less was found to be suitable for CL-MS studies without altering the protein structure [35]. Nevertheless, to avoid DEPC-induced structural perturbation of mAb samples, a 6× molar excess of DEPC was used in our labeling experiment, consistent with the published reaction kinetics study. Our optimized protocol further included TCEP as a reducing agent and involved 3 h for tryptic digestion to enable maximum DEPC label detection.

The main aim of this work was to further study the structural destabilization of mAb samples subjected to stress conditions, namely (i) heat stress; (ii) long-term storage (2–8 °C), and (iii) a combination of both. Heat stress (65 °C - onset point of structural changes according to nanoDSF analysis results) was applied to the mAb samples for a limited time of 1 h to enable observation of the initial stage structural changes. A previous study on heat stressing mAb samples at 60 °C for 1 h reported no evident changes in the mAb structure as analyzed with intact-protein MS, dynamic light scattering, and far-UV circular dichroism [36]. On the other hand, in our study, at 65 °C heat stress for 1 h, significant changes were detected in the DEPC labeling of heat-stressed samples at two positions in the Fab region (Tyr32- also encompassing the CDR region and His208) and one position (Lys342) at the intersection of the CH2 and CH3 domains. However, for the most part, the monomeric structural stability was maintained, as evidenced also by supporting SEC-UV analysis. 

In contrast, for heat-stressed one-year-old mAb (LS_H sample set), SEC-UV analysis (of DEPC-labeled intact mAb samples) suggested the presence of high-molecular-weight aggregates. The peptide analysis results considering DEPC label positions further suggested that CDR-1 may be involved in the initiation of mAb-degradation pathway for SILuMAb. CDR involvement in mAb degradation also implies reduced antigen-binding capacity for therapeutic mAbs [37]. Similar to our observation, another DEPC-based CL-MS study also reported aggregation susceptibility near the CDR of a heat-stressed mAb [38]. These examples demonstrate that insights from DEPC-CL-MS-based structural studies could be effectively used as site-specific information for optimizing the protein sequence by CDR grafting, and could thereby achieve higher mAb stability at CDR. Irrespective of the heat stress, fragmentation was evident from the SEC-UV results of one-year-stored (at 2–8 °C) mAb samples. As with our study, fragmentation as a result of storage conditions has been reported as a degradation pathway in multiple studies, but not exclusively for refrigeration-related storage [39,40].

The fragmentation observed in the abovementioned experiment (SEC-UV analysis of LS_O samples) prompted us to investigate the structural disorder by examining the disulfide bonding for mAb samples stored at 2–8 °C for 1 yr and 2 yrs in comparison to a freshly thawed mAb sample (labeled S_0yr herein). A previous SEC-based mAb long-term stability study reported minimal degradation and oxidation of tryptophans at the storage temperature of 2–8 °C [36]. In contrast, the sensitivity of DEPC CL-MS employed to study the effect of storage on SILuMAb samples could detect significant structural changes. We used a double-labeling strategy for Cys, first labeling with DEPC and then with IAA. A similar double-labeling strategy to capture all free Cys with NEM has been reported [41]. Our study revealed that DEPC-modified cysteines were exclusively detected in long-term-stored (up to 2 years at 2–8 °C) mAb samples (Figure 6). The loss of disulfide bonding due to storage coincided with, or may even have been a contributing factor in the mAb fragmentation observed in long-term-stored mAb samples. The instability of cysteines at hinge regions is of particular interest because the intra-chain disulfide bonding cysteines are usually buried in the mAb molecule and not easily accessible to labeling reagents [42]. DEPC labeling of Cys230 and Cys233 indicated that these mAb portions were solvent-exposed (i.e., had undergone unfolding). However, Cys230 was only freed in the 2-year-refrigerated mAb sample, and as a result, most of the mAb molecules are seen in the fragment peak of SEC-UV analysis. One published article indicated that mAbs primarily fragment in the hinge region involving Cys230, especially under oxidative stress. The authors furthermore showed that Cys230 did not appear as a free SH group under their specific study conditions [43]. This supports our findings for the 1-year-old sample. The 1-year-old sample seemed to reflect an intermediate fragmentation state, showing the shift from a monomer state to a fragmented state. Most interestingly, we did not detect LC Cys91, which is an unpaired Cys in SILuMAb. The fact that LC Cys91 was not detected may indicate disulfide scrambling. While interpreting these results it should be noted that, conditions inducing minimal disulfide scrambling, for example use of acidic tryptic digestion buffers [44] were not deployed in our experiment. Rather, our experimental setup had near-neutral to basic pH conditions favoring sequencing-grade trypsin activity for efficient digestion. Apart from the accounts of disulfide scrambling for an IgG1 drug product under storage for up to 4 weeks [45] and under heat stress [46], our CL-MS analysis is one of the first reports of disturbed disulfide bonding in a long-term-stored drug substance (up to 2 years).

To clarify the nature of the fragments seen in Figure 7, we also performed an RPLC-MS analysis of the same S_2yr and S_1yr samples (Supplementary Figure S3). In the control measurement for the freshly thawed mAb sample, a peak of intact deglycosylated mAb mass (≈144 kDa) was observed (Supplementary Figure S3). It should be noted that deglycosylation was a part of experimental processing before intact-protein MS analysis. The peak representing intact deglycosylated mAb was no longer present in the stored mAb samples. Rather, there were chromatographic peaks representing masses of 14 kDa and 46 kDa fragments in the S_1yr to S_2yr samples (Supplementary Figure S3). These masses were proteolytic fragments of SILuMAb, but the typical site of cleavage was deduced from the RPLC-MS data. A potential explanation for the putative fragment seen in the S_1yr and S_2yr samples could be the increased peptide bond fragmentation associated with storage-related unfolding of flexible loops in mAbs [47]. The fragmentation observed herein could also be attributed to a combination of thawing, the pH of mAb storage solution, the stability of excipient components used, and the protease activity from trace-level CHO cell protease impurity in the mAbs [40,48]. As indicated by the manufacturer, the lyophilized SILuMAb contained phosphate-buffered saline and no other stabilizing agents. Thus, the fragmentation seen in our analysis can be explained from the reports of enhanced denaturation of proteins frozen in phosphate buffer due to freezing-dependent pH shifts [49]. Further experiments would be required to clarify the nature of these fragments.

Finally, it must be noted that this study was performed with SILuMAb and may not account for structural changes in other mAbs. We emphasize here that, the number of cysteines on a mAb, the excipients used, and the microenvironment might influence the mAb structure. Therefore, aging effects seen in other types of mAbs maybe variable. However, a similar DEPC-based CL-MS labeling strategy can be applied to the study structural changes related to monoclonal antibodies, or even more interestingly, to the study of the aggregation of single-chain variable fragments or the assembly of newer antibody formats, such as bispecific antibodies. Ultimately, our results demonstrate a straightforward method of structural protein analysis using CL-MS; however, care should be taken in the design of structure-perturbing experiments [50].

## 4. Materials and Methods

### 4.1. Overview of Experiments

(i) Assessing heat-stress-induced structural changes in SILuMAb: SILuMAb stock solution (protein lyophilized in phosphate-buffered saline reconstituted in MS grade water as 1 µg/µL solution) stored at −80 °C was thawed and aliquoted into two subsets. One set (n = 2) was exposed to heat stress of 65 °C for 1 h under gentle agitation (300 rpm) before the labeling reaction. The other sample set (n = 2) was not heat-stressed, but directly DEPC-labeled. Post-DEPC-labeling, (detailed method in Section 4.3.1) all mAb samples were deglycosylated. For bottom-up MS analysis, each sample was assessed as 2 technical replicates.

(ii) Assessing heat-stress-induced structural changes in SILuMAb after one year (1 yr) of refrigeration (“non-formulated, aged sample”): A stock solution of 2 µg/µL SILuMAb sample (protein lyophilized in phosphate-buffered saline reconstituted in MS grade water) stored at −80 °C was thawed and refrigerated at 2–8 °C for 1 yr. Note: There were no preservatives or surfactants added to prevent protein degradation. “Aged” SILuMAb samples were divided into two subsets (n = 3) and further processed as heat-stressed (65 °C for 1 h) and non-heat-stressed sample subsets, as described in (i). For bottom-up analysis, each of the 3 samples were injected twice as technical replicates, making 6 samples per sample subset.

(iii) Assessing long-term-storage-related structural changes in SILuMAb: A stock solution of 2 µg/µL SILuMAb sample (protein lyophilized in phosphate-buffered saline reconstituted in MS grade water) stored at −80 °C was thawed and refrigerated as 2 µg/µL solutions at 2–8 °C for one and two years (yrs), respectively. For comparison, a SILuMAb stock solution was thawed from −80 °C, not refrigerated but instead directly used as a fresh sample. Fresh (0 yr-old), 1 year-old, and 2-year-old SILuMAb samples (n = 2) were all DEPC-labeled.

### 4.2. Antibodies, Chemicals, Enzymes, and Materials

SILuMAb universal antibody standard human (MSQC4) was purchased from Merck, Darmstadt, Germany. DEPC was obtained from Acros Organics, Geel, Belgium (Product no. 10114380). Imidazole (Product no. I2399), urea (Product no. U5378), ammonium bicarbonate (ABC; Product no. 09830), iodoacetamide (IAA) (Product no. I6125) and C18 disks- 47 mm (Product no. 66883-U) were purchased from Merck. Tris (2-carboxyethyl) phosphine (TCEP) was obtained from Alfa Aesar, Karlsruhe, Germany, and PNGaseF was purchased from Serva, Heidelberg, Germany. Sequencing grade modified trypsin (product no. V5111) was procured from Promega (Madison, WI, USA), formic acid (FA), and LC-MS grade water from VWR (Darmstadt, Germany). LC-MS grade acetonitrile (ACN) and methanol were purchased from Honeywell (Muskegon, MI, USA). Sodium chloride was obtained from Chemsolute, Renningen, Germany, and monobasic sodium phosphate from AppliChem, Darmstadt, Germany.

### 4.3. Sample Preparation for CL-MS

#### 4.3.1. Diethyl Pyrocarbonate (DEPC) Labeling

Every experiment consisted of two SILuMAb sample sets: an untreated sample set (“original mAb”) and an experiment-specific treated sample set. For each experiment, 50 µg SILuMAb was used. Treated and untreated SILuMAb samples were labeled with DEPC reagent as follows: DEPC stock solution (69 mM in ACN) prepared freshly before each experiment was diluted in water to obtain a 1.36 mM DEPC solution. SILuMAb samples were incubated with a final 0.04 mM DEPC concentration for 5 min at 37 °C under gentle agitation. To quench the reaction, 50 mM imidazole was added at a 50-fold molar excess over DEPC. For deglycosylation, 1 µL of PNGaseF (1000 U) was added to the samples and incubated at 37 °C for 1 h under gentle agitation. Then, each sample was split into one aliquot for bottom-up MS analysis (40 µg) and one aliquot for intact-protein analysis (10 µg).

#### 4.3.2. Sample Preparation for LC-MS-Based Peptide Analysis

Original and treated mAb samples were denatured using 8 M urea prepared in 50 mM ABC. Disulfide bonds were reduced with 0.79 mM TCEP at 37 °C for 30 min under gentle agitation. Samples were further alkylated with 15 mM IAA for 30 min at RT in the dark. To reduce urea concentration before digestion, samples were diluted with 50 mM ABC to a final concentration of 1 M urea. For protein digestion, trypsin was added in a ratio of 1:40 (enzyme: mAb), and all samples were incubated for 3 h at 37 °C under gentle agitation. Digestion was stopped by adding FA to a final concentration of 0.2%. Before LC-MS analysis, peptide samples were desalted using C18 StageTips, as described by Rappsilber, Mann, and Ishihama [51]. The cleaned peptide eluates were dried down completely for 4 h in a vacuum concentrator. Dried peptide samples were later reconstituted with 0.1% FA water to a final concentration of 1 µg/µL and used for LC-MS/MS analysis.

#### 4.3.3. Sample Preparation for Intact-Protein Mass Analysis

From the remaining sample containing 10 µg labeled and deglycosylated mAb, 5 µg was saved for size-exclusion chromatography coupled to a UV detector (SEC-UV), and 5 µg was saved for LC-MS intact-mass analysis.

### 4.4. Analytical Techniques

#### 4.4.1. Nanodifferential Scanning Fluorimetry

The thermal stability of the SILuMAb was determined by nanodifferential scanning fluorimetry (nanoDSF) with the Prometheus NT. Plex instrument (nanotemper). Standard capillaries were used, the excitation power was set to 30%, and the temperature was increased from 20–95 °C with a slope of 1 °C/min. The software PR. ThermControl (v2.1) was used for data acquisition, and PR. Stability Analysis (v1.1) was used for data processing.

#### 4.4.2. LC-MS/MS-Based Tryptic Peptide Analysis

For LC-MS/MS peptide analysis, a 1260 capillary HPLC system (Agilent Technologies, Waldbronn, Germany) was coupled to an Impact II QTOF mass spectrometer with an Apollo II electrospray ionization (ESI) source (Bruker Daltonics, Bremen, Germany). Reversed-phase separation was performed on an Agilent USHSN017 C18 column (customized), 5 µm particle size, 0.5 mm inner diameter, and 150 mm length using a 45 min gradient. Mass spectra were recorded in positive ion ESI mode in the mass range from 150 to 2200 *m*/*z* (details of method in Supporting Material).

#### 4.4.3. LC-MS-Based Intact-Protein Mass Analysis

Intact-protein mass analysis was performed on a 1290 Infinity UHPLC (Agilent Technologies, Waldbronn, Germany), coupled to an Impact II QTOF-MS with Apollo II ESI source (Bruker Daltonics, Bremen, Germany). Reversed-phase separation was performed on a PLRP-S column (Agilent Technologies), with 5 µm particle size, 1000 Å pore size, 1 mm inner diameter, and 50 mm length. Mass spectra were recorded in positive ion ESI mode and the mass range from 600 to 7000 *m*/*z* (Details of LC-MS method in Supplementary Material). Data were analyzed in a Compass Data analysis 4.4 (Bruker Daltonics) tool. A weighted average of DEPC label incorporation was calculated for each sample. The weighted average of labels was calculated using the following formula:

$$\mathrm{Average}\;\mathrm{label}\;\mathrm{number}=\frac{\sum_{x=1}^{n}xI_{x}^{Modified}}{\sum_{x=1}^{n}I_{x}^{Modified}+I_{0}^{Unmodified}}$$

where x is the number of DEPC modifications, I_0_ is the intensity of unlabeled peaks, and I_x_ is the intensity of labeled peaks [35].

#### 4.4.4. SEC-UV Intact-Protein Analysis

SEC-UV analysis was performed using a 1050 series HPLC system from Hewlett-Packard (now Agilent Technologies) equipped with a diode array detector. For SEC, a TSKgel SuperSW3000 column from Tosoh Bioscience (Griesheim, Germany) was used, with a 4 µm particle size, inner diameter of 4.6 mm, and 30 cm length. The mobile phase consisted of 100 mM monobasic sodium phosphate and 100 mM sodium chloride, adjusted to pH 7.0 with sodium hydroxide. SEC-UV analysis used an isocratic flow of 0.2 mL/min with a run time of 30 min. The wavelength for UV detection was 280 nm. Chromatograms were recorded and exported as data files to be further analyzed in Microsoft Excel.

### 4.5. LC-MS/MS Data Analysis and Representation

#### 4.5.1. Peptide Quantification from LC-MS/MS Data

Peptide identification was carried out with Byos Software Version: v4.5 (Protein Metrics). Precursor and fragment mass error tolerance were both set to 30 ppm. Trypsin was set as protease, with a maximum of three missed cleavages allowed. Details of the modification list and data analysis workflow are to be found in Supporting Material. The peptides in the resulting identification list were visually verified by the shape of the XIC peak and incorporation of DEPC labels in the b or y ion series of fragment ions, and only valid peptides were further tagged and sorted as true positives. Only true-positive hits were further considered for statistical analysis.

For the experiment assessing the intactness of disulfide bonds at Cys, instead of the area under the curve of XIC, the XIC % ratio was used for quantification. The % DEPC label at Cys was calculated in this case in Byos software version: v4.5 using the % XIC ratio formula below.


$$\mathrm{XIC}\;\mathrm{ratio}\;\%=\frac{{XIC}_{\_}modifiedpeptide}{{XIC}_{\_}modifiedpeptide+{XIC}_{\_}non-modifiedpeptide}$$

#### 4.5.2. Statistical Analysis of Quantitative Peptide Data

XIC areas obtained from validated peptides tagged as true-positive (Byos software) were exported, and statistical data analysis was performed in Perseus [52]. XIC intensities were log_2_-transformed, followed by the filtering of data for valid hits. In each sample set, the hits were considered valid if 50% of the replicates had a quantification value (for example, in the case of 4 replicates, at least 2 replicates needed to have no missing XIC value). Missing values were replaced with values from a normal distribution (in Perseus), and then the dataset was used for the two-sample Student’s *t*-test. Positions of peptides carrying the modified amino acid with at least a two-fold changed DEPC intensity with *p*-value < 0.05 were plotted in a volcano scatter plot using an in-house-written R script.

#### 4.5.3. Visualization of Labeled mAb as a 3D Model

The PDB file of SILuMAb was kindly provided by Dr. Andreas Evers of the antibody discovery and protein engineering department at Merck. The PDB model was loaded into PyMOL Version 2.5.4 (Schrödinger, NY, USA). The significant DEPC-modified amino acids were highlighted in the 3D mAb model, and the label-containing models were exported as images.

## 5. Conclusions

A detailed analysis of DEPC-labeled peptides with amino acid resolution can help to address questions of site-specific unfolding or aggregation in therapeutic proteins. The mapping of aggregation-prone areas is perhaps one of the most interesting outcomes of our DEPC-based CL-MS approach. Such an analysis may support predictions of the susceptibility of CDR for aggregation and function as insightful information for techniques such as CDR grafting for increasing the stability of mAb. Within the same experimental setup, DEPC-based CL-MS may additionally help in the evaluation of disulfide bonding patterns and associated mAb fragmentation. In certain scenarios, DEPC CL-MS with rigorous data analysis can serve as an alternative to the more sophisticated mass spectrometry methods, such as HDX-MS or FPOP in the analysis of structural protein perturbations.

## Data Availability

Data is contained within the article and supplementary material.

## Supplementary Materials

The following supporting information can be downloaded at: https://www.mdpi.com/article/10.3390/ph16101418/s1, Figure S1: Reaction kinetics of DEPC labeling studied with dose response curve for different peptides of SILuMAb; Figure S2: SEC-UV analysis of standard mAb; Figure S3: Chromatograms of RPLC-MS analysis for intact DEPC labeled long-term stored SILuMAb. Table S1: Logaritmised (log 2) intensity values for the sample set of heat stressed fresh SILuMAb (SH) and non-heat stressed SILuMAb (SO). Table S2: Logaritmised (log 2) intensity values for the sample set of heat stressed one year stored SILuMAb (LS_H) and non –heat stressed one year stored SILuMAb (LS_O). Table S3: Percentage DEPC incorporation at Cys for assessing the intactness of disulfide bonds in long term stored (S_2yr, S_1yr) and fresh SILuMAb- each with 3 replicates.

## Abbreviations

Covalent labeling mass spectrometry (CL-MS), diethyl pyrocarbonate (DEPC), liquid chromatography—mass spectrometry (LC-MS), amino acid (aa), hydrogen–deuterium exchange mass spectrometry (HDX-MS), lysine (Lys), histidine (His), serine (Ser), threonine (Thr), tyrosine (Tyr), SILu™Lite SigmaMAb (SILuMAb), ammonium bicarbonate (ABC), Tris (2-carboxyethyl) phosphine (TCEP), iodoacetamide (IAA), complementary determining region (CDR), extracted ion chromatogram (XIC), heavy chain (HC), light chain (LC), nanodifferential scanning fluorimetry (nanoDSF), solvent accessible surface area (SASA), reverse phase liquid chromatography–mass spectrometry (RPLC-MS), size-exclusion chromatography coupled with UV detection (SEC-UV), year (yr), hour (h).
