# Supplementary material for: Site-Specific Structural Changes in Long-Term-Stressed Monoclonal Antibody Revealed with DEPC Covalent-Labeling and Quantitative Mass Spectrometry
Source: Pharmaceuticals (Basel). 2023 Oct 5;16(10):1418. doi: 10.3390/ph16101418 (PMC10609731; doi:10.3390/ph16101418)
Supplement: Supplementary file 1 [file pharmaceuticals-16-01418-s001.zip › pharmaceuticals-2579250-supplementary.pdf]

Supplement information:

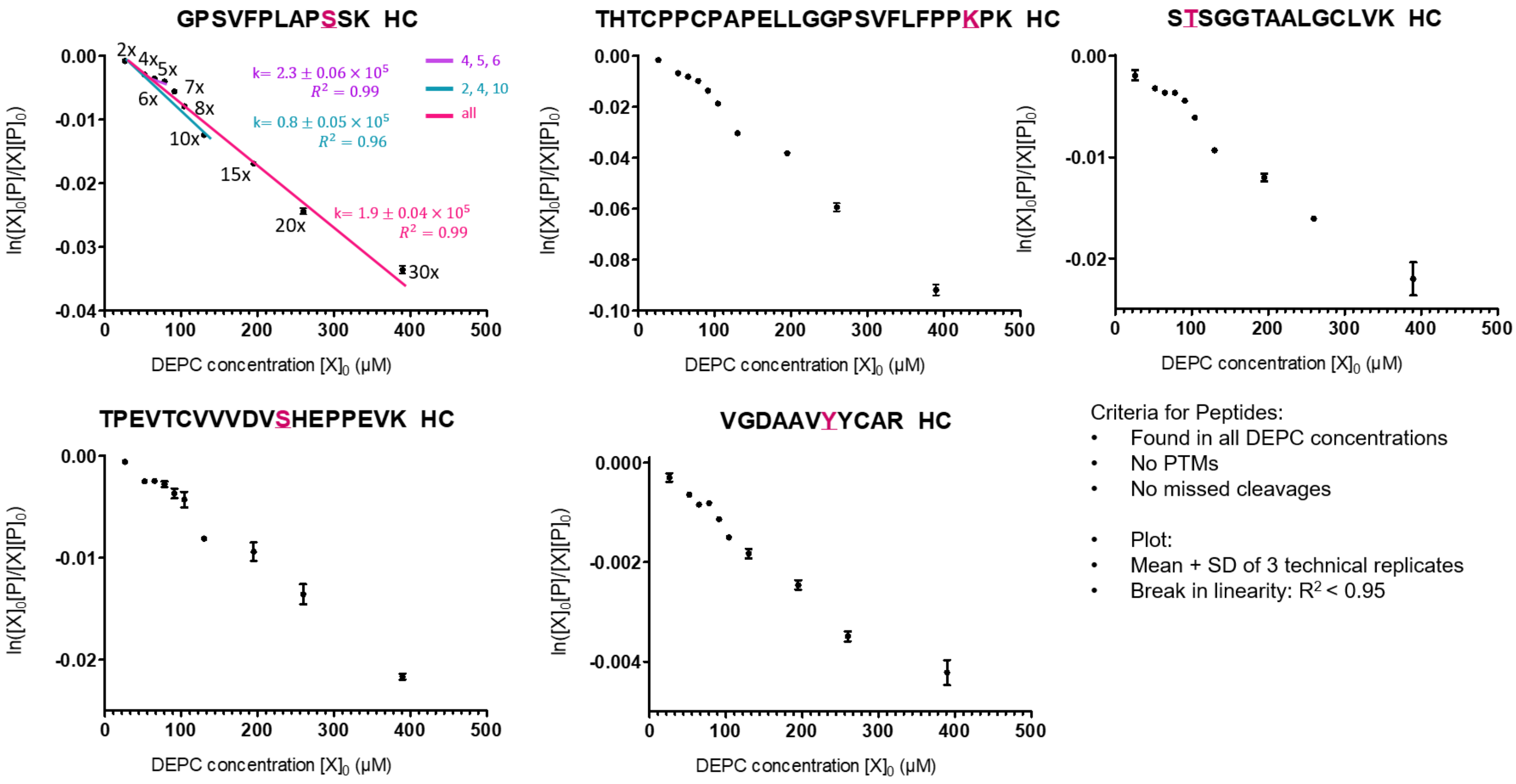

Figure S1: Reaction kinetics of DEPC labelling studied with dose response curve for different peptides of SILuMAb. The X axis is initial concentration of DEPC while Y axis is the ratio of DEPC modified peak area. Criteria for break in linearity was $R^2 < 0.95$ for the peptides assessed in our CL-MS study. HC signifies heavy chain.

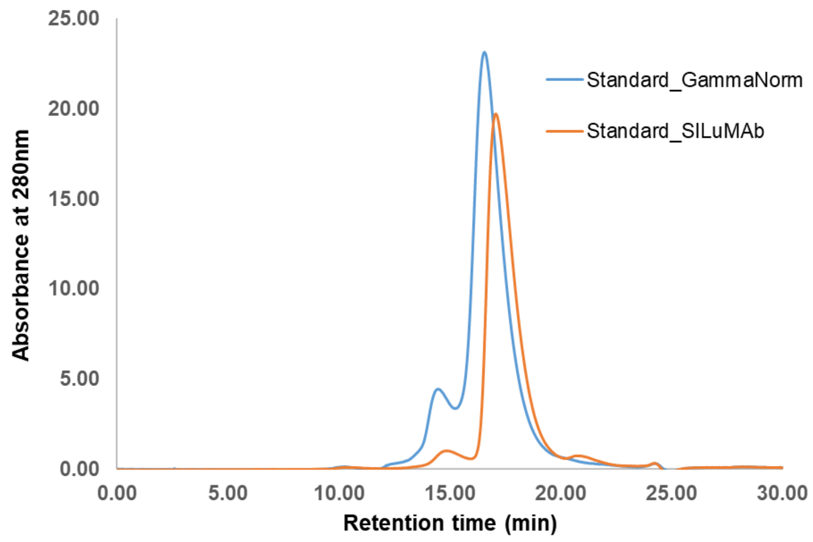

Figure S2: SEC-UV analysis of standard mAb – GammaNorm in blue and standard SILuMAb in orange. The monomer peak is seen to elute at 17-19 min, while the dimer peak is seen as small peak upfront of monomer at RT of 15 min. The standard SILuMAb also shows a minor fragment peak at 21 min. A small bump at 10 min is a column contamination visible in all samples and blanks.

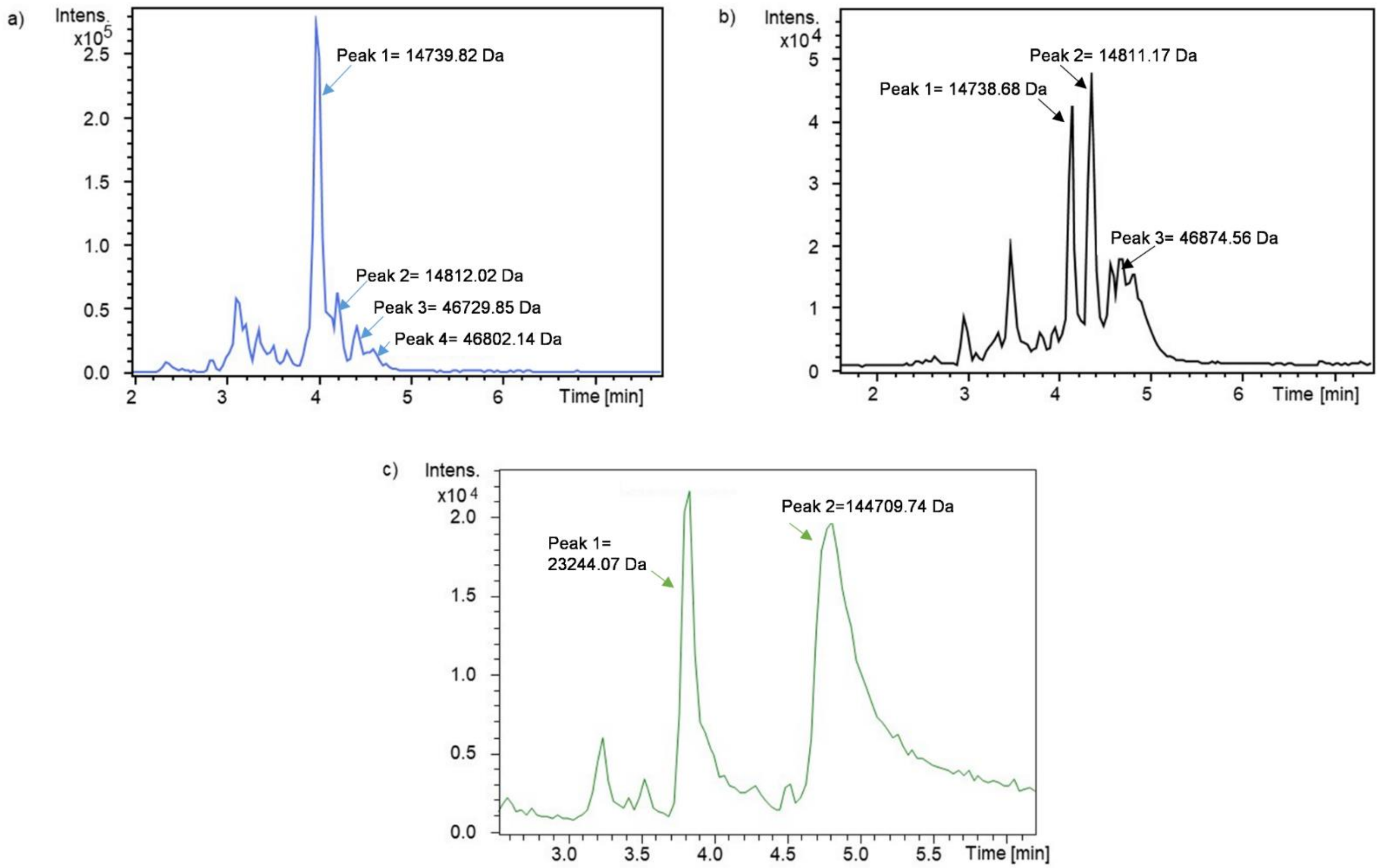

Figure S3: Chromatograms of RPLC-MS analysis for intact DEPC labeled long-term stored SILuMAb a) S_2yr sample with overhead annotation of the peak number and the mass (Da) detected at that specific RT. No monomeric peak is detected. b) S_1yr sample with overhead annotation of the peak number and the mass (Da) detected at that specific RT. Monomer mass is not detected. c) S_0yr sample with 2 peaks, and respective overhead annotations of the masses under these peaks. The second peak denotes the mass of deglycosylated and DEPC labeled monomer

Table S1: Logaritmised (log 2) intensity values for the sample set of heat stressed fresh SILuMAb (SH) and non-heat stressed SILuMAb (SO). NaN denotes missing value. The positions common with the heat stressed one-year stored SILuMAb vs non –heat stressed one year stored SILuMAb experiment (supporting table 3) are highlighted in bold & italics.

Log_2 intensity SH_1	Log_2 intensity SH_2	Log_2 intensity SH_3	Log_2 intensity SH_4	Log_2 intensity SO_1	Log_2 intensity SO_2	Log_2 intensity SO_3	Log_2 intensity SO_4	Mod. Names	Mod. AAs	AA position	Protein chain
19.95	NaN	20.68	21.07	21.21	21.22	22.28	21.55	DEPC/72.02	H	208	SILuMAb-HC
NaN	21.39	21.15	NaN	21.00	NaN	19.89	NaN	DEPC/72.02	H	272	SILuMAb-HC
NaN	***20.55***	***22.07***	***20.44***	***20.69***	***19.16***	***NaN***	***NaN***	***DEPC/72.02***	***H***	***289***	***SILuMAb-HC***
19.64	22.28	22.44	22.66	21.74	19.97	NaN	NaN	DEPC/72.02	H	314	SILuMAb-HC
21.98	***22.90***	***23.23***	***23.13***	***22.52***	***20.40***	***21.79***	***NaN***	***DEPC/72.02***	***K***	***43***	***SILuMAb-HC***
21.07	***22.37***	***21.89***	***21.80***	***21.61***	***20.97***	***20.82***	***NaN***	***DEPC/72.02***	***K***	***44***	***SILuMAb-LC***
19.37	***20.89***	***20.13***	***18.66***	***19.15***	***18.83***	***NaN***	***NaN***	***DEPC/72.02***	***K***	***47***	***SILuMAb-LC***
22.43	***23.25***	***23.02***	***22.90***	***23.53***	***22.70***	***22.92***	***22.80***	***DEPC/72.02***	***K***	***50***	***SILuMAb-HC***
22.12	***22.00***	***21.85***	***22.47***	***22.02***	***22.10***	***21.01***	***21.00***	***DEPC/72.02***	***K***	***55***	***SILuMAb-LC***

22.68	***22.91***	***23.19***	***NaN***	***23.72***	***23.75***	***22.95***	***22.97***	***DEPC/72.02***	***K***	***64***	***SILuMAb-HC***
19.97	***23.59***	***21.58***	***21.47***	***21.79***	***20.29***	***20.73***	***19.82***	***DEPC/72.02***	***K***	***75***	***SILuMAb-HC***
20.70	***21.55***	***20.70***	***21.02***	***21.22***	***19.97***	***20.69***	***NaN***	***DEPC/72.02***	***K***	***116***	***SILuMAb-LC***
19.91	21.97	NaN	NaN	21.00	NaN	19.84	NaN	DEPC/72.02	K	135	SILuMAb-LC
20.65	***20.82***	***21.53***	***20.33***	***22.37***	***21.87***	***21.20***	***21.00***	***DEPC/72.02***	***K***	***162***	***SILuMAb-LC***
19.27	19.82	21.08	21.10	20.69	20.92	19.88	18.92	DEPC/72.02	K	172	SILuMAb-LC
20.62	***21.52***	***21.83***	***21.29***	***21.11***	***NaN***	***20.67***	***NaN***	***DEPC/72.02***	***K***	***177***	***SILuMAb-LC***
21.87	***22.39***	***22.02***	***22.08***	***22.32***	***22.14***	***22.60***	***20.68***	***DEPC/72.02***	***K***	***192***	***SILuMAb-LC***
19.19	NaN	21.04	20.25	21.80	20.48	21.31	NaN	DEPC/72.02	K	209	SILuMAb-HC
NaN	22.16	22.50	NaN	23.04	19.39	NaN	18.45	DEPC/72.02	K	226	SILuMAb-HC
NaN	***20.32***	***20.81***	***21.09***	***21.34***	***NaN***	***19.63***	***NaN***	***DEPC/72.02***	***K***	***252***	***SILuMAb-HC***

22.23	***22.84***	***22.32***	***20.86***	***22.19***	***20.61***	***22.40***	***19.13***	***DEPC/72.02***	***K***	***278***	***SILuMAb-HC***
19.14	***20.83***	***19.68***	***18.95***	***20.91***	***20.54***	***19.73***	***19.08***	***DEPC/72.02***	***K***	***292***	***SILuMAb-HC***
21.84	***22.30***	***22.38***	***22.72***	***22.34***	***21.57***	***21.71***	***21.20***	***DEPC/72.02***	***K***	***321***	***SILuMAb-HC***
21.92	***22.68***	***22.01***	***22.30***	***23.28***	***21.47***	***22.28***	***NaN***	***DEPC/72.02***	***K***	***324***	***SILuMAb-HC***
NaN	20.03	20.80	NaN	21.99	21.86	21.47	NaN	DEPC/72.02	K	326	SILuMAb-HC
22.17	***23.01***	***22.32***	***NaN***	***22.45***	***22.41***	***22.32***	***21.41***	***DEPC/72.02***	***K***	***330***	***SILuMAb-HC***
23.62	***23.95***	***23.35***	***22.96***	***23.07***	***20.65***	***23.17***	***NaN***	***DEPC/72.02***	***K***	***338***	***SILuMAb-HC***
20.04	20.60	20.70	NaN	18.74	19.80	NaN	18.81	DEPC/72.02	K	342	SILuMAb-HC
21.14	21.90	22.12	NaN	22.72	22.66	NaN	21.55	DEPC/72.02	K	418	SILuMAb-HC
20.10	NaN	21.82	NaN	21.17	24.25	NaN	NaN	DEPC/72.02	S	62	SILuMAb-HC
21.21	***22.22***	***21.08***	***21.61***	***21.89***	***NaN***	***21.47***	***NaN***	***DEPC/72.02***	***S***	***135***	***SILuMAb-HC***

21.21	***22.22***	***21.93***	***21.61***	***21.89***	***20.80***	***21.47***	***19.30***	***DEPC/72.02***	***S***	***136***	***SILuMAb-HC***
18.62	19.28	18.71	NaN	19.16	NaN	19.38	19.22	DEPC/72.02	S	159	SILuMAb-LC
NaN	20.75	22.05	21.19	22.73	NaN	20.62	NaN	DEPC/72.02	S	171	SILuMAb-LC
NaN	22.65	21.25	NaN	22.63	20.76	20.86	NaN	DEPC/72.02	S	188	SILuMAb-HC
NaN	21.05	19.64	NaN	19.84	19.50	NaN	20.12	DEPC/72.02	S	198	SILuMAb-LC
19.26	***20.04***	***19.88***	***NaN***	***19.81***	***19.62***	***18.95***	***18.56***	***DEPC/72.02***	***S***	***258***	***SILuMAb-HC***
NaN	23.18	20.80	NaN	21.82	NaN	22.18	NaN	DEPC/72.02	T	191	SILuMAb-HC
20.59	21.97	21.87	20.81	19.33	NaN	18.88	NaN	DEPC/72.02	Y	32	SILuMAb-HC
NaN	***NaN***	***22.96***	***22.82***	***22.31***	***21.70***	***NaN***	***NaN***	***DEPC/72.02***	***Y***	***32***	***SILuMAb-LC***
21.27	***20.91***	***21.25***	***21.65***	***21.17***	***21.44***	***21.01***	***21.02***	***DEPC/72.02***	***Y***	***51***	***SILuMAb-LC***
NaN	NaN	19.86	19.15	NaN	18.93	20.56	NaN	DEPC/72.02	Y	183	SILuMAb-LC

20.83	***21.66***	***21.60***	***21.55***	***21.46***	***20.97***	***20.94***	***19.69***	***DEPC/72.02***	***Y***	***282***	***SILuMAb-HC***
20.79	***23.38***	***23.06***	***22.88***	***23.08***	***21.37***	***22.36***	***NaN***	***DEPC/72.02***	***Y***	***323***	***SILuMAb-HC***
NaN	19.47	20.66	20.50	21.08	NaN	18.92	21.24	DEPC/72.02;DEPC/72.02	K,S	43,49	SILuMAb-HC
20.13	21.60	21.88	22.28	22.43	20.12	21.00	NaN	DEPC/72.02;DEPC/72.02	K,S	50,62	SILuMAb-HC
NaN	NaN	19.70	20.06	20.88	19.90	NaN	NaN	DEPC/72.02;DEPC/72.02	S,K	49,64	SILuMAb-HC

Table S2: Logaritmised (log 2) intensity values for the sample set of heat stressed one year stored SILuMAb (LS_H) and non –heat stressed one year stored SILuMAb (LS_O). NaN denotes missing value. The positions common with the heat stressed fresh SILuMAb vs non –heat stressed fresh SILuMAb experiment (supporting table 2) are highlighted in bold & italics.

Log$_2$ intensity LS_H_1	Log$_2$ intensity LS_H_2	Log$_2$ intensity LS_H_3	Log$_2$ intensity LS_H_4	Log$_2$ intensity LS_H_5	Log$_2$ intensity LS_H_6	Log$_2$ intensity LS_O_1	Log$_2$ intensity LS_O_2	Log$_2$ intensity LS_O_3	Log$_2$ intensity LS_O_4	Log$_2$ intensity LS_O_5	Log$_2$ intensity LS_O_6	Mod. Names	Mod. AAs	AA position	Protein chain
23.02	24.39	24.07	22.63	24.84	23.35	23.77	23.77	23.81	23.79	23.52	23.51	DEPC/72.02	H	35	SILuMAb-HC
22.33	***22.40***	***22.10***	***21.98***	***22.76***	***22.78***	***22.68***	***22.69***	***22.93***	***22.94***	***22.66***	***22.63***	***DEPC/72.02***	***H***	***272***	***SILuMAb-HC***
21.34	***21.95***	***21.08***	***21.52***	***23.00***	***23.02***	***22.80***	***22.79***	***23.15***	***23.15***	***22.99***	***23.48***	***DEPC/72.02***	***H***	***289***	***SILuMAb-HC***
22.96	***22.95***	***22.79***	***22.72***	***23.09***	***23.36***	***23.54***	***23.52***	***23.60***	***23.54***	***23.35***	***23.70***	***DEPC/72.02***	***H***	***314***	***SILuMAb-HC***
22.59	22.23	22.84	20.88	22.90	23.15	23.94	23.48	24.23	23.86	24.30	23.35	DEPC/72.02	H	433	SILuMAb-HC
22.65	22.65	NaN	NaN	21.10	NaN	22.69	23.25	23.56	23.13	24.28	22.69	DEPC/72.02	H	437	SILuMAb-HC
22.43	NaN	NaN	22.34	24.04	21.60	NaN	21.73	21.91	23.19	NaN	23.95	DEPC/72.02	H	439	SILuMAb-HC
23.24	***23.28***	***23.31***	***23.34***	***23.80***	***23.75***	***20.55***	***20.49***	***20.84***	***20.84***	***20.65***	***20.68***	***DEPC/72.02***	***K***	***43***	***SILuMAb-HC***
22.85	***22.87***	***22.75***	***22.72***	***23.17***	***23.14***	***23.34***	***23.32***	***23.56***	***23.58***	***23.80***	***23.41***	***DEPC/72.02***	***K***	***50***	***SILuMAb-HC***
24.95	***24.94***	***24.96***	***24.96***	***25.37***	***25.34***	***25.21***	***25.21***	***25.41***	***25.40***	***26.38***	***26.36***	***DEPC/72.02***	***K***	***64***	***SILuMAb-HC***
24.94	***24.93***	***24.71***	***24.70***	***25.21***	***25.19***	***24.98***	***24.97***	***25.14***	***25.13***	***24.98***	***24.98***	***DEPC/72.02***	***K***	***75***	***SILuMAb-HC***

21.83	21.83	22.02	21.96	22.56	22.22	22.96	22.98	22.68	22.63	22.48	22.45	DEPC/72.02	K	125	SILuMAb-HC
25.06	25.05	25.06	24.53	25.39	25.13	25.16	25.17	25.07	25.46	24.44	25.38	DEPC/72.02	K	137	SILuMAb-HC
20.68	20.32	20.90	20.59	21.05	21.00	21.65	21.10	21.51	21.37	21.39	21.39	DEPC/72.02	K	250	SILuMAb-HC
21.43	***20.97***	***20.47***	***21.05***	***22.15***	***21.77***	***22.05***	***22.04***	***22.11***	***22.10***	***22.17***	***22.15***	***DEPC/72.02***	***K***	***252***	***SILuMAb-HC***
21.10	***20.92***	***20.69***	***20.83***	***21.02***	***21.02***	***21.58***	***21.52***	***21.40***	***21.49***	***21.31***	***21.44***	***DEPC/72.02***	***K***	***278***	***SILuMAb-HC***
20.34	***20.27***	***20.72***	***20.25***	***21.15***	***21.44***	***21.70***	***21.27***	***21.58***	***22.04***	***21.58***	***21.88***	***DEPC/72.02***	***K***	***292***	***SILuMAb-HC***
23.10	***23.11***	***23.44***	***23.30***	***23.64***	***23.40***	***23.35***	***23.85***	***23.21***	***23.21***	***22.96***	***22.95***	***DEPC/72.02***	***K***	***321***	***SILuMAb-HC***
22.16	***22.22***	***22.12***	***22.16***	***22.82***	***22.85***	***23.06***	***23.07***	***22.99***	***22.99***	***22.86***	***22.86***	***DEPC/72.02***	***K***	***324***	***SILuMAb-HC***
22.18	***20.90***	***NaN***	***NaN***	***22.56***	***22.38***	***NaN***	***NaN***	***23.39***	***22.77***	***NaN***	***22.70***	***DEPC/72.02***	***K***	***326***	***SILuMAb-HC***
23.35	***24.28***	***23.56***	***23.55***	***23.46***	***23.43***	***24.35***	***23.88***	***24.52***	***24.02***	***24.12***	***24.13***	***DEPC/72.02***	***K***	***330***	***SILuMAb-HC***
24.65	***24.66***	***24.97***	***24.98***	***25.26***	***25.24***	***25.67***	***25.67***	***25.82***	***25.82***	***25.81***	***25.81***	***DEPC/72.02***	***K***	***338***	***SILuMAb-HC***
NaN	***19.95***	***20.64***	***20.44***	***21.31***	***21.21***	***21.59***	***21.72***	***21.96***	***22.36***	***22.63***	***22.60***	***DEPC/72.02***	***K***	***342***	***SILuMAb-HC***
24.71	24.42	24.80	24.40	24.91	24.89	24.56	24.47	24.19	24.68	24.65	24.38	DEPC/72.02	K	364	SILuMAb-HC
22.56	22.55	22.75	22.63	23.39	23.27	24.42	23.68	23.43	23.34	23.16	23.10	DEPC/72.02	K	396	SILuMAb-HC
28.24	***28.24***	***28.30***	***28.30***	***28.63***	***28.61***	***28.41***	***28.41***	***28.55***	***28.55***	***28.57***	***28.56***	***DEPC/72.02***	***K***	***418***	***SILuMAb-HC***
21.73	NaN	23.17	23.16	NaN	23.16	NaN	23.04	23.94	NaN	23.31	23.18	DEPC/72.02	K	443	SILuMAb-HC

21.02	20.96	21.10	21.01	21.41	21.18	21.59	21.63	NaN	21.72	NaN	21.79	DEPC/72.02	S	128	SILuMAb-HC
NaN	***20.96***	***NaN***	***25.76***	***NaN***	***21.18***	***NaN***	***25.78***	***NaN***	***NaN***	***21.79***	***21.79***	***DEPC/72.02***	***S***	***135***	***SILuMAb-HC***
21.02	NaN	21.10	NaN	23.31	NaN	21.59	NaN	NaN	NaN	22.46	23.63	DEPC/72.02	S	136	SILuMAb-HC
NaN	***20.97***	***NaN***	***21.05***	***21.79***	***NaN***	***21.64***	***21.61***	***20.00***	***19.97***	***22.44***	***21.67***	***DEPC/72.02***	***S***	***258***	***SILuMAb-HC***
22.33	NaN	21.76	NaN	NaN	22.78	NaN	NaN	22.93	22.94	22.66	22.63	DEPC/72.02	S	271	SILuMAb-HC
22.35	20.33	23.39	NaN	21.14	20.87	21.30	21.32	23.02	NaN	22.26	21.95	DEPC/72.02	T	441	SILuMAb-HC
23.46	22.79	23.39	22.54	23.77	23.24	22.89	22.40	22.44	22.91	23.52	23.51	DEPC/72.02	Y	32	SILuMAb-HC
NaN	20.32	19.96	NaN	20.49	20.49	NaN	20.02	20.16	20.31	19.97	NaN	DEPC/72.02	Y	79	SILuMAb-HC
22.61	22.64	22.63	22.67	23.20	23.18	21.80	21.79	21.98	21.97	21.92	21.95	DEPC/72.02	Y	93	SILuMAb-HC
21.18	***21.29***	***21.25***	***21.21***	***21.75***	***21.81***	***21.74***	***22.16***	***22.52***	***22.53***	***22.40***	***22.39***	***DEPC/72.02***	***Y***	***282***	***SILuMAb-HC***
21.63	***21.65***	***21.86***	***21.57***	***22.14***	***22.18***	***22.56***	***22.63***	***24.22***	***24.21***	***23.98***	***23.09***	***DEPC/72.02***	***Y***	***323***	***SILuMAb-HC***
22.08	NaN	21.76	NaN	NaN	21.68	22.24	22.24	NaN	22.26	22.20	22.23	DEPC/72.02	Y	377	SILuMAb-HC
22.05	NaN	22.62	23.39	24.34	23.73	23.44	23.30	22.90	22.63	22.77	23.45	DEPC/72.02	Y	440	SILuMAb-HC
24.03	21.70	23.70	20.89	24.07	24.19	24.53	20.61	24.48	24.48	20.41	24.43	DEPC/72.02	H	41	SILuMAb-LC
NaN	23.71	23.46	23.46	23.80	23.85	23.78	23.76	NaN	24.43	NaN	24.68	DEPC/72.02	H	203	SILuMAb-LC
23.66	23.66	23.43	23.42	24.45	23.92	24.19	24.20	24.22	24.20	24.15	24.15	DEPC/72.02	K	44	SILuMAb-LC

22.69	22.71	22.78	22.76	23.18	22.85	22.53	22.47	22.69	22.64	23.07	23.06	DEPC/72.02	K	47	SILuMAb-LC
23.20	23.21	23.73	23.02	23.27	23.27	22.22	23.80	22.81	23.89	23.84	24.03	DEPC/72.02	K	55	SILuMAb-LC
21.38	21.88	21.89	NaN	NaN	NaN	22.35	22.74	22.15	22.48	NaN	22.20	DEPC/72.02	K	68	SILuMAb-LC
24.17	24.56	23.73	23.72	24.23	24.54	24.07	24.07	24.38	24.36	24.19	24.17	DEPC/72.02	K	116	SILuMAb-LC
NaN	NaN	21.61	21.51	22.27	21.48	22.66	22.64	NaN	21.98	21.74	21.60	DEPC/72.02	K	135	SILuMAb-LC
NaN	NaN	20.31	20.34	20.65	NaN	20.70	20.79	20.61	20.61	20.54	20.43	DEPC/72.02	K	155	SILuMAb-LC
23.68	23.69	23.59	23.62	24.08	24.08	23.87	23.89	23.88	23.87	24.35	24.35	DEPC/72.02	K	162	SILuMAb-LC
22.27	22.26	22.11	22.21	22.63	21.63	23.11	23.10	23.25	23.25	23.16	23.24	DEPC/72.02	K	177	SILuMAb-LC
23.73	23.70	23.93	23.54	24.04	24.07	24.48	24.14	24.04	24.36	24.42	24.42	DEPC/72.02	K	192	SILuMAb-LC
23.74	23.75	23.38	23.36	23.82	23.78	23.80	23.82	23.99	23.98	24.15	24.15	DEPC/72.02	K	210	SILuMAb-LC
NaN	NaN	20.00	20.04	20.36	20.44	19.97	20.04	20.70	21.41	22.23	21.74	DEPC/72.02	K	218	SILuMAb-HC
21.22	21.23	23.60	20.89	23.94	20.27	24.40	24.44	24.32	24.38	24.25	24.42	DEPC/72.02	Y	32	SILuMAb-LC
24.86	20.53	24.56	24.57	20.84	25.21	25.30	21.49	25.43	21.44	21.42	25.34	DEPC/72.02	Y	38	SILuMAb-LC
23.15	23.14	23.50	23.49	23.66	23.66	23.73	24.11	24.30	24.30	24.00	24.43	DEPC/72.02	Y	51	SILuMAb-LC
22.65	22.59	22.64	22.64	22.09	22.39	22.04	22.01	21.83	22.17	22.92	22.85	DEPC/72.02; DEPC/72.02	H,K	314, 321	SILuMAb-HC
22.46	NaN	22.34	NaN	23.18	21.15	21.67	22.83	22.75	NaN	NaN	21.27	DEPC/72.02; DEPC/72.02	H,K	314, 324	SILuMAb-HC

22.81	21.88	22.98	22.36	22.00	24.31	22.46	23.84	23.66	23.34	24.10	23.56	DEPC/72.02; DEPC/72.02	H,Y	433, 440	SILuMAb-HC
21.76	21.34	20.10	NaN	20.44	24.10	24.30	20.51	NaN	22.48	23.13	NaN	DEPC/72.02; DEPC/72.02	H,Y	437, 440	SILuMAb-HC
22.02	22.49	NaN	NaN	22.42	24.28	22.21	NaN	22.01	24.47	24.46	NaN	DEPC/72.02; DEPC/72.02	H,Y	439, 440	SILuMAb-HC
22.19	22.20	22.62	21.77	22.66	22.64	22.74	22.77	22.77	22.76	22.39	22.42	DEPC/72.02; DEPC/72.02	K,S	43,49	SILuMAb-HC
24.55	24.52	24.98	24.20	22.92	NaN	25.20	24.75	25.26	25.24	24.56	25.69	DEPC/72.02; DEPC/72.02	K,S	50,62	SILuMAb-HC
20.81	22.54	21.30	21.29	21.78	21.78	22.94	21.79	22.09	NaN	22.84	22.13	DEPC/72.02; DEPC/72.02	K,S	324, 328	SILuMAb-HC
25.52	25.52	24.54	25.26	25.48	24.76	25.41	25.42	25.50	NaN	25.48	25.94	DEPC/72.02; DEPC/72.02	K,S	338, 341	SILuMAb-HC
21.71	21.67	21.82	21.83	22.18	22.11	21.90	21.89	21.91	21.92	21.97	21.96	DEPC/72.02; DEPC/72.02	K,S	418, 419	SILuMAb-HC
21.53	21.38	21.13	20.93	22.15	22.01	21.77	21.98	21.85	21.61	21.33	21.49	DEPC/72.02; DEPC/72.02	K,T	250, 254	SILuMAb-HC
22.67	22.69	22.99	23.01	22.59	23.03	23.13	23.40	23.58	23.56	23.54	22.76	DEPC/72.02; DEPC/72.02	K,T	330, 339	SILuMAb-HC
22.14	22.16	21.82	21.83	22.42	22.40	21.56	21.95	22.10	21.77	21.40	21.37	DEPC/72.02; DEPC/72.02	K,Y	75,79	SILuMAb-HC
21.02	21.00	20.97	20.93	21.85	21.82	21.39	21.38	21.19	21.22	21.36	21.33	DEPC/72.02; DEPC/72.02	K,Y	321, 323	SILuMAb-HC
20.54	20.55	20.64	20.66	20.94	20.96	NaN	20.31	20.77	20.74	20.21	20.59	DEPC/72.02; DEPC/72.02	S,K	223, 226	SILuMAb-HC
24.44	24.44	24.42	24.40	24.76	24.76	24.38	24.38	24.50	24.50	24.55	24.56	DEPC/72.02; DEPC/72.02	S,K	341, 344	SILuMAb-HC
20.57	20.56	NaN	20.48	21.33	21.89	21.52	21.47	21.54	21.48	21.33	21.24	DEPC/72.02; DEPC/72.02	Y,K	323, 324	SILuMAb-HC
22.31	22.39	NaN	22.16	22.82	22.85	22.94	22.98	22.86	22.85	23.31	22.50	DEPC/72.02; DEPC/72.02	Y,K	323, 326	SILuMAb-HC

23.36	21.83	22.97	22.99	23.49	23.48	22.28	24.34	23.58	23.53	23.36	23.33	DEPC/72.02; DEPC/72.02	H,K	41,44	SILuMAb-LC
20.93	21.39	NaN	NaN	NaN	21.80	21.95	21.98	NaN	20.99	20.69	21.07	DEPC/72.02; DEPC/72.02	H,K	41,47	SILuMAb-LC
NaN	NaN	22.88	22.83	23.35	23.20	23.50	NaN	22.95	22.94	22.74	22.70	DEPC/72.02; DEPC/72.02	K,H	192,194	SILuMAb-LC
22.33	NaN	22.32	NaN	22.81	NaN	NaN	22.13	NaN	22.41	22.37	NaN	DEPC/72.02; DEPC/72.02	K,S	47,58	SILuMAb-LC
22.71	22.79	21.94	22.55	22.45	23.27	22.65	22.68	22.90	22.68	22.71	23.37	DEPC/72.02; DEPC/72.02	K,S	162,171	SILuMAb-LC
24.09	24.82	24.57	22.58	25.08	24.39	24.97	24.19	23.86	24.39	24.55	24.31	DEPC/72.02; DEPC/72.02	K,S	177,193	SILuMAb-LC
21.74	NaN	22.32	22.33	22.81	22.72	22.14	22.13	NaN	22.41	22.37	22.34	DEPC/72.02; DEPC/72.02	K,T	47,54	SILuMAb-LC
20.63	20.69	20.53	NaN	21.84	21.24	21.27	21.98	21.40	21.62	NaN	21.07	DEPC/72.02; DEPC/72.02	K,Y	44,51	SILuMAb-LC
21.52	21.52	21.30	21.65	22.05	21.45	22.11	22.08	22.30	21.61	21.50	22.26	DEPC/72.02; DEPC/72.02	K,Y	47,51	SILuMAb-LC
20.87	NaN	20.64	NaN	21.25	NaN	21.22	21.19	21.25	NaN	21.10	21.05	DEPC/72.02; DEPC/72.02	K,Y	177,178	SILuMAb-LC
21.99	22.02	21.56	NaN	NaN	22.16	NaN	22.22	NaN	22.12	21.92	21.96	DEPC/72.02; DEPC/72.02	S,K	36,44	SILuMAb-LC
20.08	NaN	20.50	20.47	NaN	NaN	NaN	21.32	21.48	22.41	22.86	22.77	DEPC/72.02; DEPC/72.02	S,K	158,162	SILuMAb-LC
NaN	21.27	21.06	20.99	NaN	NaN	21.81	NaN	21.86	21.86	NaN	20.52	DEPC/72.02; DEPC/72.02	S,K	185,192	SILuMAb-LC
20.55	20.48	21.36	21.22	21.10	21.06	21.56	21.55	21.91	22.08	22.38	22.72	DEPC/72.02; DEPC/72.02	T,K	151,162	SILuMAb-LC
24.40	23.70	23.34	24.01	23.83	23.83	24.65	23.90	24.56	23.75	23.61	23.58	DEPC/72.02; DEPC/72.02	Y,K	32,44	SILuMAb-LC
23.70	23.70	23.34	23.35	NaN	23.83	NaN	23.21	23.79	23.75	23.61	23.58	DEPC/72.02; DEPC/72.02	Y,K	38,44	SILuMAb-LC

22.63	NaN	22.51	22.50	22.94	22.91	22.65	22.68	22.93	22.91	22.93	22.13	DEPC/72.02; DEPC/72.02	Y,K	51,55	SILuMAb-LC
21.21	20.66	20.36	21.02	20.95	NaN	21.10	NaN	NaN	21.20	21.87	21.13	DEPC/72.02; DEPC/72.02	Y,K	178,192	SILuMAb-LC
21.21	NaN	23.22	23.62	24.19	23.88	23.49	23.48	24.32	23.77	23.60	NaN	DEPC/72.02; DEPC/72.02	Y,K	183,192	SILuMAb-LC
23.10	23.46	23.07	23.07	23.46	23.38	23.64	23.64	23.62	23.98	22.91	22.90	DEPC/72.02; Oxidation/15.99	K,M	75,82	SILuMAb-HC
NaN	21.14	20.95	20.96	NaN	21.38	NaN	20.99	21.03	21.32	NaN	20.47	DEPC/72.02; Oxidation/15.99	Y,M	32,34	SILuMAb-HC
20.84	20.89	21.07	20.98	21.30	20.86	21.82	21.82	21.94	21.96	21.44	21.43	DEPC/72.02; Oxidation/15.99	K,M	47,49	SILuMAb-LC
21.58	NaN	NaN	23.83	NaN	24.31	NaN	23.98	25.57	23.98	NaN	24.83	DEPC/72.02; urethane-CEt histidine/134.06	H,H	433,437	SILuMAb-HC
24.13	23.34	23.94	23.09	23.60	24.41	24.02	24.85	24.67	23.25	24.02	25.53	DEPC/72.02; urethane-CEt histidine/134.06	H,H	433,439	SILuMAb-HC
NaN	NaN	24.04	23.74	25.26	NaN	NaN	NaN	NaN	23.78	25.24	24.83	DEPC/72.02; urethane-CEt histidine/134.06	H,H	437,439	SILuMAb-HC
NaN	20.49	20.48	20.42	20.76	NaN	NaN	20.22	NaN	21.16	20.38	20.36	DEPC/72.02; urethane-CEt histidine/134.06	K,H	226,228	SILuMAb-HC
22.84	22.81	22.74	22.75	23.15	24.17	23.05	NaN	23.16	23.15	23.24	23.24	DEPC/72.02; urethane-CEt	S,H	430,439	SILuMAb-HC

												histidine/134.06			
22.42	21.68	21.23	21.90	22.10	21.96	21.38	NaN	21.15	21.08	20.68	20.58	DEPC/72.02; urethane-CEt histidine/134.06;DEPC/72.02	H,H,Y	433, 439, 440	SILuMAb-HC
20.44	20.47	20.60	NaN	20.92	20.85	20.39	20.46	20.40	20.33	20.44	20.36	Gln->pyro-Glu/-17.02;DEPC/72.02	Q,S	1,2	SILuMAb-LC
22.01	22.03	22.66	21.97	22.52	22.06	22.71	23.16	22.69	22.67	22.77	23.86	Oxidation/15.99;DEPC/72.02	H,K	228, 252	SILuMAb-HC
20.23	20.27	20.11	NaN	NaN	20.49	21.22	21.22	20.97	20.98	20.45	20.38	Oxidation/15.99;DEPC/72.02	M,K	116, 125	SILuMAb-HC
NaN	22.02	21.80	21.79	21.69	22.36	21.91	21.92	22.20	22.20	21.81	21.79	Oxidation/15.99;DEPC/72.02	M,K	49,55	SILuMAb-LC
21.78	21.80	21.96	21.98	22.15	22.24	22.23	21.96	22.42	22.02	21.98	21.61	Oxidation/15.99;DEPC/72.02	M,Y	49,51	SILuMAb-LC
21.21	21.21	20.86	NaN	NaN	20.69	22.10	22.13	21.77	NaN	NaN	21.19	Oxidation/15.99;DEPC/72.02	W,K	37,44	SILuMAb-LC
22.90	22.78	21.15	22.66	21.24	21.24	21.18	21.11	21.19	21.19	20.80	20.81	Oxidation/15.99;DEPC/72.02;urethane-CEt histidine/134.06	M,H,H	432, 433, 439	SILuMAb-HC

20.08	20.18	20.76	20.02	20.56	21.04	20.76	20.92	20.98	20.96	20.97	20.93	urethane-CEt histidine/134.06;DEPC/72.02	H,K	228, 250	SILuMAb-HC
21.36	21.39	NaN	NaN	NaN	21.84	22.39	22.40	22.25	22.25	22.34	20.04	urethane-CEt histidine/134.06;DEPC/72.02	H,K	228, 252	SILuMAb-HC
20.44	20.42	20.70	20.22	21.23	20.76	21.17	21.22	NaN	21.05	NaN	21.13	urethane-CEt histidine/134.06;DEPC/72.02	H,K	314, 321	SILuMAb-HC
20.24	20.19	NaN	NaN	20.76	NaN	21.62	21.59	21.29	21.27	21.24	21.15	urethane-CEt histidine/134.06;DEPC/72.02	H,K	314, 324	SILuMAb-HC
22.84	NaN	22.74	22.75	23.15	24.50	23.04	23.06	NaN	24.11	23.24	22.47	urethane-CEt histidine/134.06;DEPC/72.02	H,T	437, 441	SILuMAb-HC
23.78	24.42	22.80	22.63	24.17	23.28	NaN	23.62	NaN	24.30	24.17	22.87	urethane-CEt histidine/134.06;DEPC/72.02	H,Y	437, 440	SILuMAb-HC
NaN	23.88	24.26	23.74	24.21	24.20	NaN	23.72	23.80	23.78	23.76	23.75	urethane-CEt histidine/134.06;DEPC/72.02	H,Y	439, 440	SILuMAb-HC
22.04	22.01	21.28	21.17	21.94	21.99	21.41	21.45	21.90	21.88	21.50	21.50	urethane-CEt histidine/134.06;DEPC/72.02	H,K	41,44	SILuMAb-LC

22.03	22.07	21.39	NaN	22.33	21.21	22.55	22.56	22.18	22.20	21.90	21.88	urethane-CEt histidine/134.06;DEPC/72.02;DEPC/72.02	H,K,T	228, 250, 254	SILuMAb-HC

Table S3: Percentage DEPC incorporation at Cys for assessing the intactness of disulfide bonds in long term stored (S_2yr, S_1yr) and fresh SILuMAb- each with 3 replicates.

SILuMAb-HC	Cys position	S_2yr (1)	S_2yr (2)	S_2yr (3)	S_2yr (4)	S_1yr (1)	S_1yr (2)	S_1yr (3)	S_1yr (4)	S_0yr (1)	S_0yr (2)	S_0yr (3)	S_0yr (4)
	22	1.46			1.4	2.14							
	95	2.29	2.55	2.41	2.09	2.94	2.95						
	148	15	15.2	14.7	14.6	16.6	16.9	18.4	19.1		33.9		
	230		14.7	13.9	14.1		16.5						
	233	14.7	15.2	13.9	14	18.1	16.5		20.6				
	265	8.55	4.39	4.55	4.73	7.33	4.96	6.3	10.1				
	429				23.7	25.9		28.2	26.2				
SILuMAb-LC	199	30.6	21.2	21.4	16.2	21.8	16.3	64.4	66.8				

Supporting method 1: Peptide cleanup using C18 stagetips:

In brief, two layers of C18 solid phase extraction material were manually tightly filled into 100 µL pipette tips. The C18 material was activated with 100 µL methanol and then 100 µL 0.1% FA in 80% ACN. The tips were centrifuged after each step at 2000 x g for 2 min or until the solutions completely passed through the C18 phase. For equilibration, the tips were washed with 100 µL 0.1% FA in water (twice), and liquid was drained by centrifugation. Next, 200 µL of tryptic digest was added at a time to the prepared tips and centrifuged until the whole sample was loaded. StageTips were washed twice with 100 µL 0.1% FA water. The C18 disk-bound tryptic peptides were eluted with 100 µL of 0.1% FA in 80% ACN.

Supporting method 2: Detailed method for LC-MS based tryptic peptide analysis

Mobile phase A consisted of 0.1% FA in water, mobile phase B of 0.1% FA in ACN. Column oven temperature was set to 40 °C and flow rate was 12 µL/min. The gradient (total time 45 min) was as follows: 2 min 5% B, then within 30 min up to 40% B, then up to 90% B within 1 min, keeping it for 4 min at 90% B, and finally down to 5% B within 1 min. The column is re-equilibrated for 7 min at 5% B. For the ESI source, end plate offset was 500 V, capillary offset 4500 V, nebulizer gas flow 0.7 bar, dry gas flow 6L/min, and dry temp 200 °C. Auto MS/MS scan mode was applied with the following parameters: cycle time 2s, threshold per 1000, sum 500 cts, fixed MS/MS acquisition 0.68 s. For each sample, two technical replicates were recorded.

Supporting method 3: Detailed method for LC-MS based intact protein mass analysis

Mobile phase A consisted of 0.1% FA in water, mobile phase B of 0.1% FA in ACN. Column oven temperature was set to 80 °C and flow rate was 0.35 mL/min. The gradient was as follows: 20% B at 4 min, 40% B at 5min, 70% B at 10 min, 90% B at 11 min, 20% B at 12 min, 20% B at 15 min. For the ESI source, end plate offset was 500 V, capillary offset 4500 V, nebulizer gas flow 3 bar, dry gas flow 12 L/min, and dry temp. 300 °C and in-source CID 120 eV

Supporting method 4: Detailed data analysis method for quantification of peptides from LC-MS/MS data

Data analysis was performed in Byos software from Protein metrics. For identification of peptides, the modification list included carbamidomethylation (57 Da) as fixed modification in most experiments. Only for the long-storage experiment, carbamidomethylation was set to variable modification. Otherwise, common variable modifications included pyroglutamate formation at the protein N terminus (-17.02 Da), oxidation (15.99 Da) at Met, carbethoxy modification with DEPC reagent (72.02 Da) at His, Lys, Cys, Ser, Thr, and Tyr. Neutral losses of DEPC from Lys and His (46.04 Da) or neutral loss from Ser and Thr (90.03 Da), were set as rare variable modifications. To allow maximum identification of DEPC modified peptides, other rare modifications considered were urethane carbethoxy histidine (134.06 Da), formyl carbethoxy histidine (162.05 Da) and di- carbethoxy histidine (146.06 Da). A total of four modifications (two of common and two of rare modifications) were allowed per peptide. Results were filtered to only include peptides with 2-7 charges, a peptide score of >150, delta modification score >10 and extracted ion chromatogram (XIC) area >3e5.
